# Gut microbiome and serum metabolome alterations associated with lactose intolerance (LI): a case‒control study and paired-sample study based on the American Gut Project (AGP)

**DOI:** 10.1128/msystems.00839-24

**Published:** 2024-09-25

**Authors:** Hong Xue, Yitian Wang, Chunfeng Mei, Lili Han, Mengxiong Lu, Xuan Li, Ting Chen, Fengyun Wang, Xudong Tang

**Affiliations:** 1 Digestive Laboratory of Traditional Chinese Medicine, Research Institute of Spleen and Stomach Diseases, Xiyuan Hospital, China Academy of Chinese Medical Sciences, Beijing, China; 2 Department of Integrated Traditional Chinese and Western Medicine, Peking University Health Science Center, Beijing, China; 3 Department of Gastrointestinal Medicine, Peking University Traditional Chinese Medicine Clinical Medical School (Xiyuan), Beijing, China; The University of Hong Kong, Hong Kong, Hong Kong, China

**Keywords:** lactose intolerance, gut microbiome, serum metabolome, metagenomics, 16S rRNA, fecal microbiota transplantation (FMT)

## Abstract

Lactose intolerance (LI) is a prevalent condition characterized by gastrointestinal symptoms that arise following lactose consumption. Recent evidence suggests that the gut microbiome may influence lactose levels in the gut. However, there is limited understanding regarding the alterations in microbiota and metabolism between individuals with LI and non-LI. This study conducted a paired-sample investigation utilizing data from the American Gut Project (AGP) and performed metagenomic and untargeted metabolomic analyses in a Chinese cohort to explore the interaction between the gut microbiome and serum metabolites. In addition, fecal microbiota transplantation (FMT) experiments were conducted to further examine the impact of the LI-associated gut microbiome on inflammatory outcomes. We identified 14 microbial genera that significantly differed between LI and controls from AGP data. Using a machine learning approach, group separation was predicted based on seven species and nine metabolites in the Chinese cohort. Notably, increased levels of *Escherichia coli* in the LI group were negatively correlated with several metabolites, including PC (22:6/0:0), indole, and Lyso PC, while reduced levels of *Faecalibacterium prausnitzii* and *Eubacterium rectale* were positively correlated with indole and furazolidone. FMT-LI rats displayed visceral hypersensitivity and an altered gut microbiota composition compared to FMT-HC rats. Metagenomic and metabolomic analyses revealed an enrichment of MAPK signaling in LI, which was confirmed by FMT-LI rats showing higher expression of ERK and RAS, along with increased concentrations of proinflammatory cytokines. This study provides valuable insights into the disrupted microbial and metabolic traits associated with LI, emphasizing potential microbiome-based approaches for its prevention and treatment.

**IMPORTANCE:**

Lactose intolerance (LI) is a prevalent condition characterized by gastrointestinal symptoms after lactose consumption due to a deficiency of lactase. There is limited understanding regarding the microbiota and metabolic alterations between individuals with LI and non-LI. This study represents the first exploration to investigate metagenomic and metabolomic signatures among subjects with lactose intolerance as far as our knowledge. We identified 14 microbial genera in the Western cohort and 7 microbial species, along with 9 circulating metabolites in the Chinese cohort, which significantly differed in LI patients. Metagenomic and metabolomic analyses revealed an enrichment of MAPK signaling in LI patients. This finding was confirmed by FMT-LI rats, exhibiting increased expression of ERK and RAS, along with higher concentrations of pro-inflammatory cytokines. Our study provides insights into the disrupted functional and metabolic traits of the gut microbiome in LI, highlighting potential microbiome-based approaches for preventing and treating LI.

## INTRODUCTION

Lactose intolerance (LI) is a common condition characterized by gastrointestinal symptoms such as bloating, borborygmi, flatulence, abdominal pain, and diarrhea, which occur following the consumption of food containing lactose, a disaccharide (1). LI can affect a significant percentage of the population, with prevalence rates ranging from 33% to 75%. These rates vary across different racial and ethnic groups, with Europeans showing the lowest occurrence and populations of Asian, Native American, and African descent displaying a higher prevalence (1–3). In addition to these gastrointestinal issues, studies have shown that individuals with lactose intolerance also have an increased risk of developing various extra-intestinal diseases, including certain types of cancers (4).

The malabsorption of lactose is caused by a deficiency of lactase, an enzyme found in the brush border of the small intestinal mucosa. Unabsorbed lactose enters the colon and is then fermented by colonic bacteria. The fermentation process produces short-chain fatty acids, primarily acetate, propionate, and butyrate, as well as gas (1). A reduction in the capacity for bacterial fermentation in the colon leads to decreased production of short-chain fatty acids. Consequently, the ability of the colon to absorb water and electrolytes is reduced, resulting in osmotic diarrhea (5). In addition to lactase deficiency, bacterial fermentation ability also contributes significantly to lactose intolerance. Lactose intolerance is a consequence of the interaction between the host genome and the gut microbiota (6). The LCT gene, which encodes the lactase enzyme, is associated with the abundance of Bifidobacterium in lactase-nonpersistent (LNP) individuals (7). Bifidobacterium species are known to metabolize lactose (8–10). Lactose, a unique carbohydrate found in most mammalian milk and consumed as part of the human diet, can positively impact the gut microbiota and promote the growth of Bifidobacteria and other lactose-fermenting bacteria (11–13). These findings emphasize the interconnectedness between the gut microbiome and the metabolism of lactose in the host. Thus, the development of lactose intolerance involves complex interactions between the gut microbiota and lactose fermentation.

Variations in the gut microbiota composition can indicate differences in lactose fermentation ability, which can affect the host’s metabolic status through the production of metabolites. Recent research has demonstrated associations between changes in the gut microbiota, its metabolites, and human health, including the development of various diseases (14–17). The extent to which LI and healthy controls (HCs) demonstrate discernible patterns in gut microbial communities and metabolic characteristics remains unclear. Hence, it is imperative to thoroughly investigate the characteristics of the gut microbiota and serum metabolism in individuals with LI. To ensure a more comprehensive analysis, we chose two cohorts from diverse regions, namely, the USA and China, considering the influence of race and ethnicity on the prevalence of lactose intolerance.

Our primary objective was to compare the composition of the gut microbiota between individuals with and without LI. To achieve this goal, we analyzed the gut microbiota compositions of 1,124 participants who self-reported either lactose intolerance (562) or nonlactose intolerance (562) using sequencing data from the AGP. We employed a one-to-one pairing algorithm to match an equal number of control participants. In addition, to examine the functional characteristics of the gut microbiota and metabolism specifically associated with lactose intolerance in China, where LIs are known to be prevalent, we conducted integrative metagenomic and metabolomic analyses on paired fecal and serum samples. This analysis aided in the identification of a specific set of serum metabolites associated with lactose intolerance. Furthermore, we developed a random forest classifier based on the predicted metagenome function and evaluated its performance using independent data sets. To gain further insight, we constructed and compared bacterial genus coabundance networks between the HC and LI groups. Furthermore, we conducted an FMT experiment and established a LI rat model to deepen our understanding of how the gut microbiota and its enriched metabolic pathways interact with lactose-intolerant hosts. This approach allowed us to investigate the effects of gut microbiota alterations on LI development and progression.

## MATERIALS AND METHODS

### Data availability

This study utilized the open database of AGP gut microbe samples, which was created by the American Gut Consortium in November 2012 (18). Samples collected internationally were shipped domestically to aggregation sites and stored at −80°C until they were transported to the United States. The samples were subjected to 16S rRNA sequencing (V4 region) using the Illumina MiSeq, Illumina HiSeq Rapid Run, and Illumina HiSeq High-Output platforms. The raw fastq files were obtained from the European Bioinformatics Institute (EBI) database under project number PRJEB11419. Consent was obtained from all participants under approved Institutional Review Board protocols: University of Colorado Boulder (protocol no. 12–0582; December 2012 to March 2015) or University of California, San Diego (protocol no. 141853; February 2015 to present) (18). No personally identifiable data were included in the public database or accessed in the present study (18).

### Building cohorts with fully matched confounders for the LI and on-LI groups

A total of 562 individuals self-reporting LIs and 562 individuals self-reporting non-LIs were included in the study. A comprehensive list of the host variables involved is provided in Table S1. Following the criteria established in a previous study (19), we included data from individuals with LI and without LI, while excluding those who were younger than 18 years, older than 80 years, had a BMI less than 12.5 or greater than 40, resided outside the United States, the United Kingdom, and Canada, did not provide fecal samples, lacked data on bowel movement quality, had taken antibiotics within 6 months, or had type 2 diabetes. The controls consisted of 6,487 individuals chosen based on the same criteria from a pool of 25,830 individuals who did not exhibit symptoms of LI. The fully matched algorithm was devised based on previously identified microbiota-confounding variables, which included BMI, sex, age, geographical location, frequency of alcohol consumption, and dietary intake of meat/eggs, dairy, vegetables, whole grains, and salted snacks (19). Pairwise Euclidean distances were calculated between the LI patients and HCs using the above-mentioned set of matching variables, which were normalized to have a mean of zero and a variance of one (centered and scaled). We conducted one-to-one pairing using the R code accessible at https://github.com/ivanvujkc/AGP_confounders.

### Processing and interpretation of 16S rRNA sequence data

Raw fastq files were processed using QIIME 2 (20). Deblurring was utilized for read denoising and generating an amplicon sequence variant (ASV) table. Taxonomy was annotated using the gg-13-8-99-515-806-nb classifier in QIIME 2 (21). A considerable number of low-abundance features may increase computational demands and potentially affect FDR-corrected *P* values when comparing variations in high-abundance outcomes, leading to potential false negatives. To prevent such errors, we excluded sequences from the feature table that were present in fewer than 30 samples and had an absolute abundance of less than 10 reads. Alpha diversity metrics, including the Shannon index, richness, Chao1, and ACE, as well as beta diversity metrics, such as the Bray‒Curtis, Unifrac, Jaccard, and Aitchison distance, were computed using R software (version 4.2.2). The Galaxy web application (http://galaxy.biobakery.org/) (22) and ImageGP (23) were utilized to identify potential metagenomic biomarkers through linear discriminant analysis effect size (LEfSe). PICRUSt2 (version 2.5.2) (24) and STAMP (25) were used to predict functional profiles for KEGG pathways.

### A prospective machine learning model for identifying LI

To assess the potential of gut microbiota parameters as markers for LI diagnosis using data from the AGP, we employed decision trees, support vector machine (SVM), random forest, K-nearest neighbors (KNN), XGBoost, and various ensemble techniques to identify the most effective algorithms for achieving accurate prediction outcomes. The area under the curve (AUC) was used to evaluate the performance of the constructed models. Subsequently, we constructed the most effective models again using metagenomes and the combination of metagenomes and metabolomes as training and validation sets, respectively. Shapley Additive exPlanations (SHAP) is a method of explainable AI utilized to clarify the outcomes generated by a machine learning model. This method was employed to identify both positive and negative correlations between biomarkers and the LI.

### Case‒control cohort

Participants were diagnosed with LI based on the results of the hydrogen breath test. The exclusion criteria included individuals who had used antibiotics, steroid hormones, or probiotics within the past 3 months. In addition, individuals with active gastrointestinal diseases, liver cirrhosis, cancer, diabetes, or ongoing infections were excluded from the study. HCs with hypertension, diabetes, obesity, or metabolic syndrome and pregnant individuals were excluded from the study. Notably, HCs were required to report no discomfort following the consumption of dairy products and exhibit negative results in the hydrogen breath test.

### Stool and plasma sample collection

Fecal samples were collected from all 82 recruited participants. Stool samples were rapidly frozen in liquid nitrogen and stored at −80°C until further use. Elbow vein blood (3 mL) was drawn from 28 patients with LI and 28 HCs between 6 a.m. and 7 a.m. in a fasting state using vacutainer tubes containing heparin. Subsequently, the blood was centrifuged immediately for 10 min at 3500 rpm and 4°C. Each aliquot of plasma sample (1.5 mL) was stored at −80°C until ultrahigh-performance liquid chromatography coupled with mass spectrometry (LC/MS) analysis was conducted.

### DNA extraction

Total genomic DNA was extracted from 0.5 g of stool material with the PF Mag-Bind Stool DNA Kit (Omega Biotek, Norcross, GA, U.S.) according to the manufacturer’s instructions. The concentration and purity of the extracted DNA were determined using TBS-380 and NanoDrop2000 spectrophotometers, respectively. DNA quality was assessed using a 1% agarose gel.

### Metagenomic sequencing

The DNA extract was fragmented to an average size of approximately 400 bp using the Covaris M220 system (Gene Company Limited, China) to facilitate paired-end library construction. Paired-end libraries were constructed using the NEXTFLEX Rapid DNA-Seq Kit (Bioo Scientific, Austin, TX, USA). Paired-end sequencing was performed on an Illumina NovaSeq 6000 (Illumina Inc., San Diego, CA, USA) at Majorbio Bio-Pharm Technology Co., Ltd. (Shanghai, China) using a NovaSeq 6000 S4 Reagent Kit according to the manufacturer’s instructions (https://www.illumina.com/). We used Diamond (version 2.0.13, available at https://github.com/bbuchfink/diamond) to align the amino acid sequences of the non-redundant gene set with the NR database (BLASTP alignment parameters set with an e-value of 1e−5). Species annotation was obtained through the taxonomic information database corresponding to the NR database, and the abundance of each species was calculated by summing the gene abundances corresponding to that species. The metagenomic sequencing data associated with this project have been deposited in the NCBI Sequence Read Archive Database under accession number SRP492714.

### Processing of metagenome sequencing data

Data analysis was conducted on the Majorbio Cloud Platform, a free online platform accessible at https://www.majorbio.com/. Initially, raw sequencing reads underwent adaptor trimming, and low-quality reads (length < 50 bp, quality value < 20, or containing N bases) were eliminated using fastp (26) (version 0.20.0), available at https://github.com/OpenGene/fastp. Alignment of reads to the human genome was performed using BWA (27) (version 0.7.17), which is accessible at http://biobwa.sourceforge.net, and any corresponding hits and their mate reads were excluded. The quality-filtered data were assembled using MEGAHIT (28) (version 1.1.2), which is available at https://github.com/voutcn/megahit. Contigs with a length of ≥300 bp were retained for the final assembly. Prodigal (29) (version 2.6.3), available at https://github.com/hyattpd/Prodigal, was utilized to predict open reading frames (ORFs) from each assembled contig. ORFs with a length of ≥100 bp were extracted. A nonredundant gene catalog was generated using CD-HIT (30) (version 4.7), accessible at https://github.com/weizhongli/cdhit, with 90% sequence identity and 90% coverage thresholds. Gene abundance for individual samples was estimated using SOAP aligner (31) (version soap2.21 release), available at https://github.com/ShujiaHuang/SOAPaligner, with a threshold of 95% identity.

### Taxonomic and functional annotation

Nonredundant genes were taxonomically classified by alignment against the NCBI NR database using DIAMOND (32) (version 2.0.11) with an e-value cutoff of 1e−5. Functional annotation, including gene ontology (GO), Kyoto Encyclopedia of Genes and Genomes (KEGG), clusters of orthologous groups (COG), and carbohydrate-active enzymes (CAZy), was also performed for these genes. Differential analysis was conducted at the taxonomic, functional, or gene-wise levels based on the taxonomic and functional annotation as well as the abundance profile of nonredundant genes using *t*-tests and Kruskal‒Wallis tests.

### Metabolite extraction

To extract metabolites, 65 µL of the liquid sample was combined with 195 µL of a solution consisting of acetonitrile and methanol at a 1:1 ratio (vol:vol) containing 0.02 mg/mL of the internal standard (L-2-chlorophenylalanine) in a 1.5 mL centrifuge tube. The mixture was vortexed for 30 s and then subjected to low-temperature sonication for 30 min at 5°C and 40 kHz. Next, the samples were incubated at −20°C for 30 min to precipitate proteins. Subsequently, the samples were centrifuged for 15 min at 4°C and 13,000 × *g*. The resulting supernatant was carefully removed and dried under nitrogen. The dried sample was then reconstituted with a 100 µL solution of acetonitrile and water at a 1:1 ratio, followed by extraction via low-temperature ultrasonication for 5 min at 5°C and 40 kHz. After centrifugation at 13,000 × *g* and 4°C for 10 min, the supernatant was transferred to sample vials for LC‒MS/MS analysis.

### Quality control sample

As part of the system conditioning and quality control (QC) procedures, a pooled QC sample was created by combining equal volumes of all individual samples. The QC samples underwent disposal and testing in the same manner as the analytical samples. This process facilitated the representation of the entire sample set and allowed for regular monitoring of analysis stability by injecting QC samples at regular intervals, typically every 5–15 samples.

### UHPLC-–MS/MS analysis

LC‒MS/MS analysis of the sample was conducted using a Thermo UHPLC-Q Exactive HF-X system equipped with an ACQUITY HSS T3 column (100 mm × 2.1 mm i.d., 1.8 µm; Waters, USA) at Majorbio Bio-Pharm Technology Co., Ltd. (Shanghai, China). The mobile phases comprised 0.1% formic acid in water:acetonitrile (95:5, vol/vol) (solvent A) and 0.1% formic acid in acetonitrile:isopropanol:water (47.5:47.5, vol/vol) (solvent B). For positive ion mode separation, the gradient was as follows: 0–3 min, mobile phase B increased from 0% to 20%; 3–4.5 min, mobile phase B increased from 20% to 35%; 4.5–5 min, mobile phase B increased from 35% to 100%; 5–6.3 min, mobile phase B was maintained at 100%; 6.3–6.4 min, mobile phase B decreased from 100% to 0%; and 6.4–8 min, mobile phase B was maintained at 0%. For negative ion mode separation, the gradient was as follows: 0–1.5 min, mobile phase B increased from 0% to 5%; 1.5–2 min, mobile phase B increased from 5% to 10%; 2–4.5 min, mobile phase B increased from 10% to 30%; 4.5–5 min, mobile phase B increased from 30% to 100%; 5–6.3 min, mobile phase B was maintained at 100%; 6.3–6.4 min, mobile phase B decreased from 100% to 0%; and 6.4–8 min, mobile phase B was maintained at 0%. The flow rate was set to 0.40 mL/min, and the column temperature was maintained at 40°C.

MS conditions: The mass spectrometric data were acquired using a Thermo UHPLC-Q Exactive HF-X mass spectrometer equipped with an electrospray ionization (ESI) source operating in both positive and negative modes. The optimal conditions were set as follows: source temperature set to 425°C; sheath gas flow rate maintained at 50 arb; auxiliary gas flow rate set to 13 arb; ion-spray voltage floating (ISVF) at −3,500 V in negative mode and 3,500 V in positive mode; and normalized collision energy adjusted from 20 V to 60 V in a rolling manner for MS/MS. The full MS resolution was set to 60,000, and the MS/MS resolution was set to 7,500. Data acquisition was performed in Data Dependent Acquisition (DDA) mode, covering a mass range from 70 to 1,050 *m*/*z*.

### Data analysis

LC/MS raw data preprocessing was executed using Progenesis QI software (Waters Corporation, Milford, USA), resulting in a three-dimensional data matrix exported in CSV format. This matrix included sample information, metabolite names, and mass spectral response intensities. Internal standard peaks and known false-positive peaks, such as noise, column bleeds, and derivatized reagent peaks, were removed, the data matrix was dereplicated, and the peaks were pooled. Simultaneously, metabolites were identified by querying databases, primarily HMDB (http://www.hmdb.ca/), Metlin (https://metlin.scripps.edu/), and the Majorbio Database.

The database-generated data matrix was uploaded to the Majorbio cloud platform (https://cloud.majorbio.com) for further analysis. Initially, the data matrix underwent preprocessing as follows: a minimum of 80% of metabolic features detected in any sample set were retained. Subsequently, for specific samples with metabolite levels below the lower limit of quantification, the minimum metabolite value was estimated, and each metabolic signature was normalized to the sum. To mitigate errors stemming from sample preparation and instrument instability, sample mass spectrometry peak intensities were normalized using the sum normalization method, yielding the normalized data matrix. In addition, variables of QC samples with a relative standard deviation (RSD) exceeding 30% were excluded and log10-transformed to obtain the final data matrix for subsequent analysis.

Next, the R package “ropls” (version 1.6.2) was utilized to conduct principal component analysis (PCA) and orthogonal least partial squares discriminant analysis (OPLS-DA), with seven cycles of interactive validation to assess model stability. Metabolites with variable importance in projection (VIP) score exceeding 1 and a *P* value less than 0.05, as determined by Student’s *t*-test, were considered significantly different. Differentially abundant metabolites between groups were then mapped to biochemical pathways through metabolic enrichment and pathway analysis based on the KEGG database (http://www.genome.jp/kegg/). These metabolites were classified according to the pathways they were involved in or the functions they performed. Enrichment analysis was employed to ascertain whether a group of metabolites appeared in a specific functional node. The Python package “scipy.stats” (https://docs.scipy.org/doc/scipy/) was used to perform enrichment analysis, elucidating the most relevant biological pathways for experimental treatments.

### Coabundance network analysis

We initially selected the top 100 gut microbiota species based on SHAP analysis from a sample comprising 82 individuals with LI. At the species level, we identified 55 differentiated species that were present in >20% of the samples for microbial network.

Using the R programming language, Spearman correlation coefficients were computed for each bacterium in both the LI and HC groups. Subsequently, the resulting *P* values were adjusted using the Benjamini‒Hochberg false discovery rate (FDR < 0.05). We then employed the weighted gene coexpression network analysis (WGCNA) method to convert the similarity matrix into modules. These modules were subsequently utilized as input for cluster analysis via the Ward clustering algorithm, resulting in the identification of 10 coabundance groups (CAGs). To visualize the coabundance networks, Cytoscape 3.9.1 software was used. Next, Wilcoxon rank-sum tests were conducted to examine statistically significant differences in relative abundance within each clustered CAG between individuals with LI and HCs. GraphPad Prism 8.3.0 was used to visualize the statistical results.

### FMT experiment in an antibiotic-treated rat model

Twelve male Wistar rats (180 ± 20 g; SPF Beijing Vital River Laboratory Animal Technology Co., Ltd.) were randomly divided into control and experimental groups and allowed to acclimate for 1 week prior to the experiments. The rats were housed under specific pathogen-free conditions with *ad libitum* access to food and water and maintained at 22°C under a 12:12 h light/dark cycle with 55–60% humidity (*n* = 6 rats per group).

The rats received filter-sterilized water supplemented with ampicillin (1 g/L), metronidazole (1 g/L), neomycin sulfate (1 g/L), and vancomycin (0.5 g/L) (Aladdin, Shanghai, China) for 2 weeks to establish the antibiotic-treated rat model. Fresh fecal samples from three individuals with LI and three HCs were randomly collected weekly and stored at −80°C. Fecal suspensions were prepared by combining samples from three subjects within the same group, as previously described. Subsequently, 2 mL of the mixed fecal suspension was administered via oral gavage to each antibiotic-treated rat for seven consecutive days. Body weights, fecal pellet numbers, abdominal withdrawal reflex (AWR) scores, and pain thresholds were recorded. Visceral sensitivity to colorectal distention (CRD) was assessed using the AWR score, with behavioral responses measured by a blinded observer. The AWR was graded as follows: 0 for normal behavior without response; 1 for slight head twist; 2 for abdominal muscle contraction; 3 for abdominal wall lifting; and 4 for body arching and lifting of the pelvic structures. At the end of week 4, the rats were sacrificed, and blood samples and tissues were collected and snap-frozen. The fecal pellets were collected and quickly stored at −80°C.

Proximal colonic tissues were obtained from both groups. Colonic tissue segments (0.5 cm) were fixed in a 4% paraformaldehyde solution for at least 24 h. Following dehydration and transparency procedures, the tissues were embedded in paraffin and sliced continuously at 3 µm for subsequent immunohistochemical staining.

### DNA extraction and PCR amplification

Total microbial genomic DNA was extracted from rat fecal samples using the FastPure Stool DNA Isolation Kit (MJYH, shanghai, China) according to the manufacturer’s instructions. The quality and concentration of DNA were determined by 1.0% agarose gel electrophoresis and a NanoDrop ND-2000 spectrophotometer (Thermo Scientific Inc., USA) and kept at −80°C prior to further use. The hypervariable regions V3-V4 of the bacterial 16S rRNA gene were amplified with primer pairs 338F (5′-ACTCCTACGGGAGGCAGCAG-3′) and 806R(5′-GGACTACHVGGGTWTCTAAT-3′) by a T100 Thermal Cycler (BIO-RAD, USA). The PCR mixture including 4 µL 5 × Fast Pfu buffer, 2 µL 2.5 mM dNTPs, 0.8 µL each primer (5 µM), 0.4 µL Fast Pfu polymerase, 10 ng of template DNA, and ddH2O to a final volume of 20 µL. PCR amplification cycling conditions were as follows: initial denaturation at 95°C for 3 min, followed by 27 cycles of denaturing at 95°C for 30 s, annealing at 55°C for 30 s and extension at 72°C for 45 s, and single extension at 72°C for 10 min, and end at 4°C. All samples were amplified in triplicate. The PCR product was extracted from 2% agarose gel and purified. Then quantified using Synergy HTX (Biotek, USA).

### 16S rRNA gene amplicon sequencing

Deep sequencing was performed on the MiSeq platform at Allwegene Company (Beijing). After the run, image analysis, base calling, and error estimation were performed using Illumina Analysis Pipeline Version 2.6. The raw sequencing reads were deposited into the NCBI Sequence Read Archive (SRA) database (accession number: SRP514692).

### Immunohistochemical analysis of RAS, ERK and NF-κB

The sections were deparaffinized in xylene and then hydrated in a graded alcohol/water bath. Antigen retrieval was performed by subjecting the sections to EDTA (pH 8.0) in a pressure cooker for 2.5 min. Subsequently, endogenous peroxidase activity was blocked by incubating the sections in endogenous hydrogen peroxide blockers (Maixin Biotechnology, SPKIT-A3, Fuzhou, China) for 10 min. Further blocking was carried out using 3% H_2_O_2_ for an additional 10 min. Primary antibodies targeting Ras, p44/42 MAPK (Erk1/2), and NF-κB p65 (Cell Signaling Technology, 67648, 8242, 4695, USA) were applied to the sections, followed by incubation in wet boxes for 1.5 h. Afterward, the sections were washed three times with PBS (10 min each) and incubated with secondary antibodies (Maixin Biotechnology, MaxVision TM HRP-Polymer Anti-Mouse/Rabbit IHC Kit, KIT-5030, Fuzhou, China) at room temperature for 25 min. DAB chromogen [Maixin Biotechnology, DAB Kit (20×), DAB-0031, Fuzhou, China] was then added and incubated at room temperature for 8 min. Five typical fields of view (100× magnification) were randomly captured from each section. The average optical density (MOD) of each image was measured using ImageJ software (33) (Java-based image processing and analysis program of public domain developed by Wayne Rasband NIH, Bethesda, MD, USA). The average MOD values obtained from the five images of each pathological section were used to represent the mean optical density values of the specimens. The expression intensity of the antibodies is expressed as the mean ± standard deviation of the mean optical density (x ± s).

### ELISA

Following lysis in lysis buffer, the colonic samples were centrifuged at 5,000 rpm for 15 min, and the supernatants were collected for analysis. Blood samples were obtained from the abdominal aorta, and after centrifugation of the whole blood at 3,000 × *g* for 10 min, the medicated serum was isolated. The levels of IL-6 and IL-1β were determined by ELISA in accordance with the manufacturer’s instructions.

### Statistical analysis

R software version 4.2.2 was utilized for conducting the diversity analysis. Differences between the two groups were assessed using paired sample *t*-tests with Prism software (version 8.0.0, SAS Institute, Inc.). Principal coordinate analysis (PCoA) and nonmetric multidimensional scaling (NMDS) were employed to visualize the clustering of samples based on their relative bacterial abundance via PERMANOVA. LEfSe, including the Kruskal‒Wallis test, Wilcoxon rank-sum test, and linear discriminant analysis, was utilized for conducting differential analysis.

## RESULTS

### Characterization of the fully paired cohorts and gut microbiota signatures of AGP

**Fig 1:**
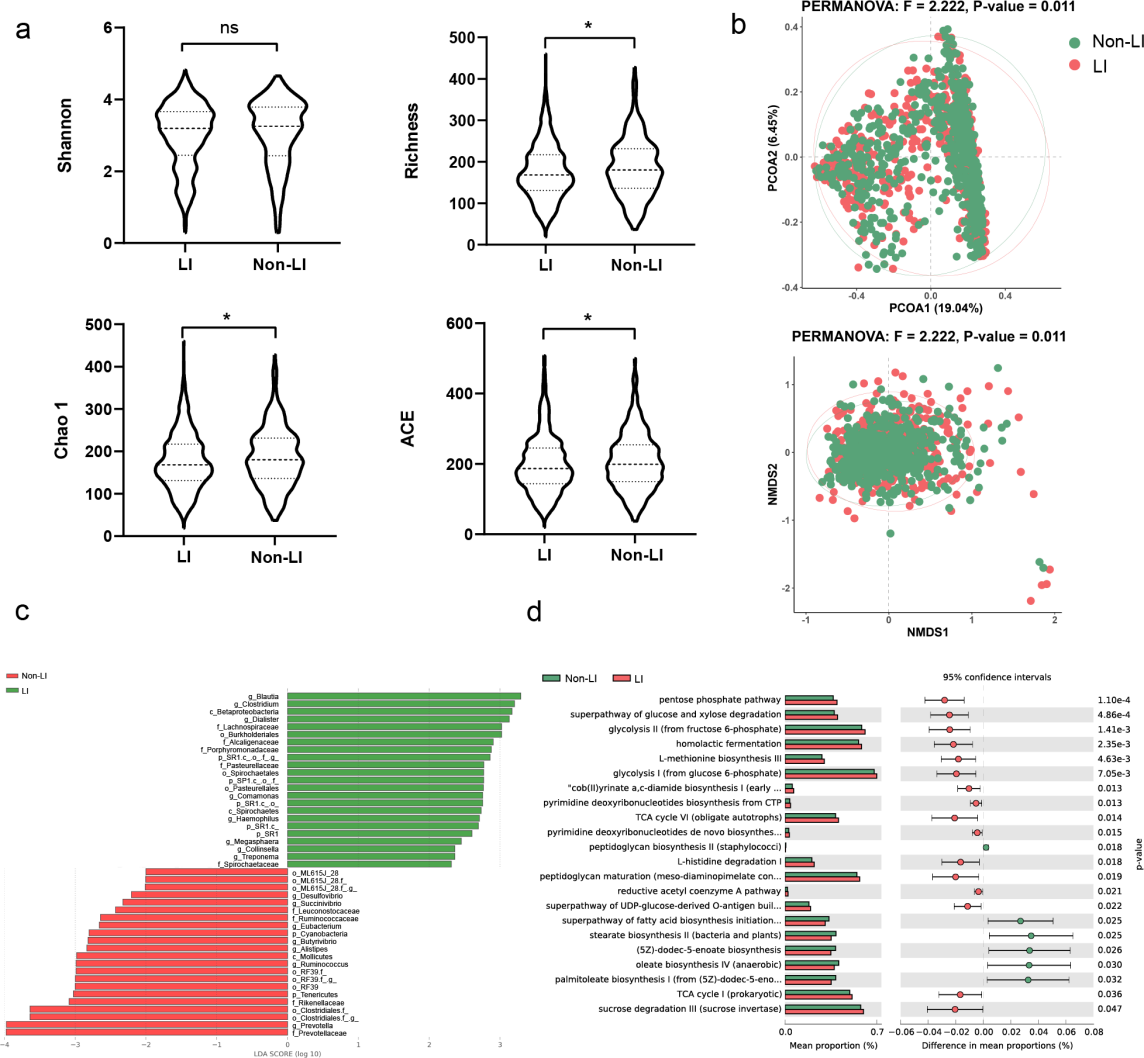
Characteristics and functional alterations in the gut microbiota in AGP. (a) The alpha diversity (Shannon index, Richness, Chao1, and ACE) of the gut microbiota in patients with and without LI; ns *P* > 0.05, *P < 0.05. (b) PCoA and NMDS of the gut microbiota composition of individuals in the LI and non-LI groups based on Bray‒Curtis dissimilarities. (c) Gut microbiota alterations were assessed by comparing the LI cohort and non-LI cohort using the linear discriminative analysis (LDA) effect size (LEfSe) biomarker discovery tool (*P* < 0.05, LDA > 2). (d) Predicting differences in KEGG pathways using PICRUSt2 (*P* < 0.05).

Before the matching procedure, a total of 895 individuals with LI were identified based on self-reported LI questionnaires. After one-to-one pairing, we included data from 562 self-reported LI patients and 562 self-reported non-LI individuals for subsequent data analysis. The baseline characteristics of the matched samples are presented in Table S1. No significant differences were detected between the LI group and their paired controls in terms of BMI, sex, age, geographical location, alcohol consumption, or frequency of dietary intake of meat/eggs, dairy, vegetables, whole grains, or salted snacks (*P* > 0.05, Fig. S1). We compared the species diversity of α-diversity between the LI and non-LI groups. Common alpha diversity metrics, including the Shannon, richness, Chao1, and ACE indices, were calculated (Fig. 1a). No difference in the Shannon index was observed between the LIs and controls (Fig. 1a, upper left). Compared with the controls, the LI group exhibited a significant decrease in bacterial richness (*P* < 0.001, paired samples *t*-test, Fig. 1a, upper right). The median Chao1 and ACE values were significantly lower in the LI patients than in their corresponding non-LI patients (*P* < 0.001, *n* = 1,124, paired samples *t*-test, Fig. 1a, lower). Furthermore, we analyzed the differences in β-diversity between the two groups. PCoA of the Bray–Curtis dissimilarities and NMDS revealed that the gut microbiota composition of the LI patients was distinct from that of the non-LI patients (*P* < 0.05, PERMANOVA test, Fig. 1b).

Subsequently, we conducted LEfSe analysis to compare the taxonomic and functional signatures between the two groups. Based on the linear discriminative analysis (LDA) scores, individuals with LI exhibited significantly greater relative abundances of Blautia, Clostridium, and Betaproteobacteria, while the non-LI microbiome was predominantly characterized by Prevotellaceae, Prevotella, etc. (LDA score > 2) (Fig. 1c). To further investigate the changes in metabolic pathways between LIs and non-LIs, we used the bioinformatics tool Phylogenetic Investigation of Communities by Reconstruction of Unobserved States (PICRUSt2) to predict microbial gene function. With a significance level (corrected) of less than 0.05, a total of 22 terms were identified. Figure 1d shows abnormalities in the pentose phosphate pathway, the superpathway of glucose and xylose degradation, glycolysis II (from fructose 6-phosphate), homolactic fermentation, and L-methionine biosynthesis III, which may be closely associated with the occurrence of LI. The pentose phosphate pathway, a superpathway of glucose and xylose degradation, showed notable differences between the LI and non-LI groups, with the LI group exhibiting the highest abundance. Similarly, peptidoglycan biosynthesis II (staphylococci), the superpathway of fatty acid biosynthesis initiation (*E. coli*), and stearate biosynthesis II (bacteria and plants) were most significantly pronounced in the non-LI group.

### Identification and validation of biomarkers associated with LI in AGP

**Fig 2:**
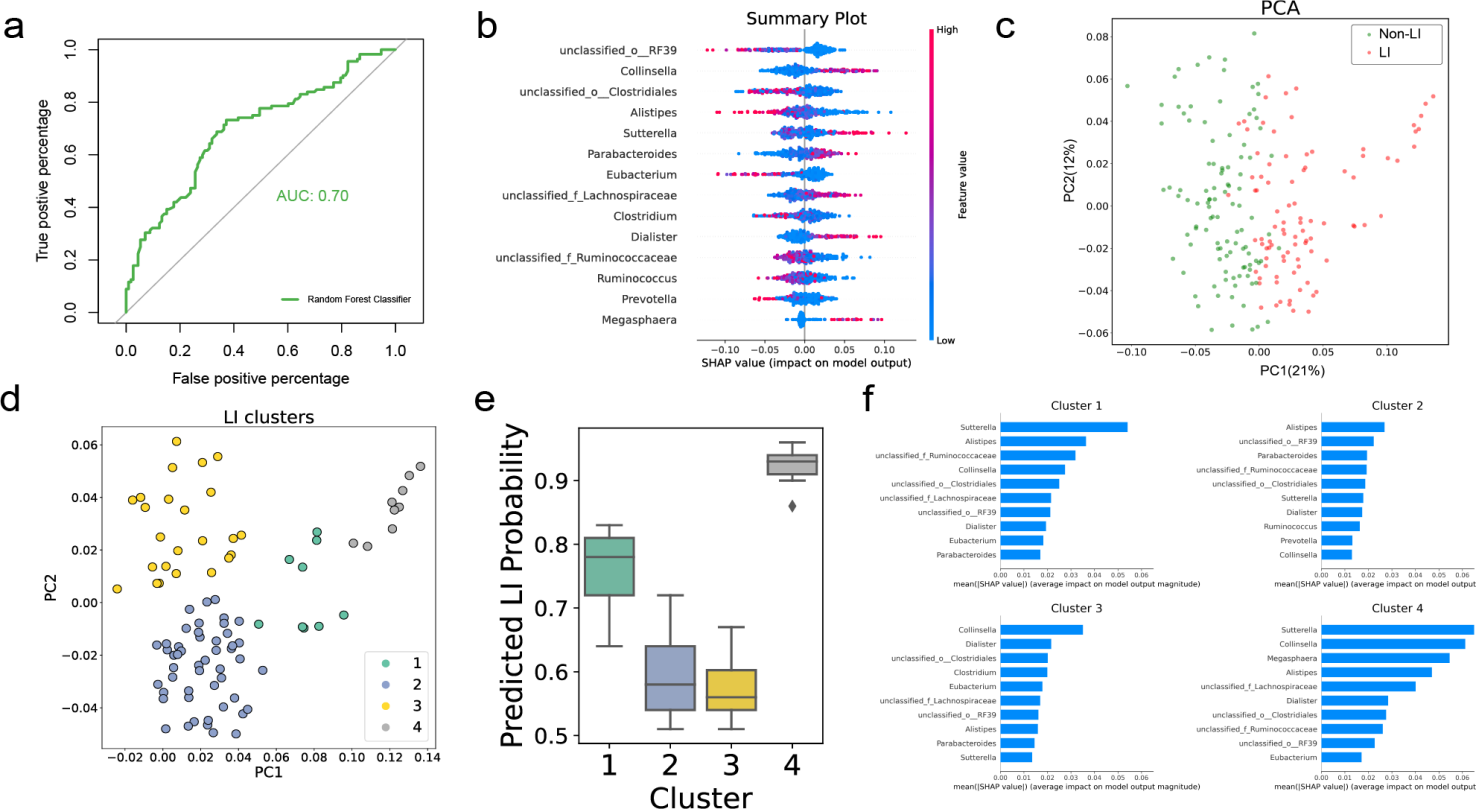
Predictive performance of machine learning models in AGP. (a) The AUC values of the random forest models. (b) This beeswarm plot provides a summary of SHAP values for all subjects, with each dot representing a specific value for an individual. The bacteria are arranged from top to bottom based on their mean absolute SHAP values, representing their global feature importance. Dots with high relative abundance (colored in red) are positioned on the positive side of the *x*-axis, signifying that a higher abundance of these bacteria is associated with an increased likelihood of LI, and vice versa. (c) PCA plots of the relative abundance data. The subjects in the plots are distinguished by color, with blue dots indicating HC participants and red dots representing those with LI. (d) K-means clustering of LI subjects. (e) Boxplot of the LI probability. (f) Feature importance of each cluster.

To assess the potential of utilizing microbial genera as effective biomarkers for characterizing the microbial community of LIs, machine learning was implemented to classify individuals with LIs and without LIs. The training and testing sets are split at a 7:3 ratio. The 1,124 individuals of AGP were randomly divided into two subsets: training (consisting of 393 individuals with LI and 393 non-LI) and testing (consisting of 169 individuals with LI and 169 non-LI). Tenfold cross-validation techniques were employed during the analysis. Based on the outcomes of the random forest model, the best combination of thresholds was found to be 1e-07 (abundance) and 0.95 (prevalence). The performance of the random forest classifier was evaluated by calculating the AUC. The random forest algorithm exhibited the strongest predictive capability among the 10 models, with an AUC of 0.70 (Fig. 2a; Fig. S2a). Furthermore, we employed the SHAP framework to enhance the interpretability of the results by visualizing the 14 microbial genera identified by random forest. To present a comprehensive overview, the SHAP values for all individuals are presented in a collection of beeswarm plots, collectively known as the summary plot (Fig. 2b). The presence of most red dots on the positive side of the *x*-axis (Collinsella, Sutterella, Parabacteroides, g_unclassified Lachnospiraceae, Dialister, and Megasphaera) suggested a correlation between the high abundance of these bacteria and a greater incidence of LI. Conversely, when the blue dots were mostly located on the positive side of the *x*-axis (g_unclassified RF39, g_unclassified Clostridiales, and Alistipes), the result was reversed. Subsequently, PCA was conducted based on the SHAP values (Fig. 2c), revealing a noticeable separation between LI and non-LI along PC1. The scatter plot was color-coded according to the predicted LI probabilities, and the results showed that individuals positioned to the right of PC1 often exhibited higher LI probabilities. Given the clear separation between LI and non-LI subjects, along with a consistent trend of LI probability, it is possible to cluster the LI data points into subgroups and hypothesize that these subgroups would exhibit similar LI probabilities (Fig. 2c). To achieve this, K-means clustering was performed on PC1 and PC2 of the SHAP values belonging to the LI subjects in each data set (Fig. 2d). The optimal number of clusters, as determined by the elbow method, was found to be 4 (Fig. S2b), as shown in Fig. 2e, demonstrating the highest median LI probability compared to the other clusters. Furthermore, Fig. 2f visualizes the feature importance for each cluster, revealing that cluster 4 predominantly consisted of bacteria such as Sutterella, Collinsella, Megasphaera, Alistipes, g_unclassified Lachnospiraceae, Dialister, g_unclassified_Clostridiales, g_unclassified Ruminococcaceae, g_unclassified RF39 and Eubacterium.

### Comparison of metagenomic and metabolomic signatures between LI patients and HCs in China

Forty-one patients with LI and HCs were recruited from Xiyuan Hospital. The detailed characteristics are presented in Table S2. We conducted shotgun metagenomic sequencing on the feces of the LI group, which were diagnosed by hydrogen exhalation, and the HC group to investigate whether there were differences in the gut microbial structures between the two groups. The ɑ-diversity measurements, including the richness and Shannon and Simpson indices, showed no significant differences (Wilcoxon rank-sum test, *P* > 0.05) at the species and genus levels between the two groups (Fig. S3a). PCoA analysis based on Bray‒Curtis distance comparisons at the NR level indicated no significant community dissimilarities between the groups (Fig. S3b). However, the groups could be distinctly separated based on PCoA analysis using the binary Jaccard algorithm for the COG functional genes (Fig. S3c). This finding suggested significant differences in the functions of the intestinal microbes between the two groups.

**Fig 3:**
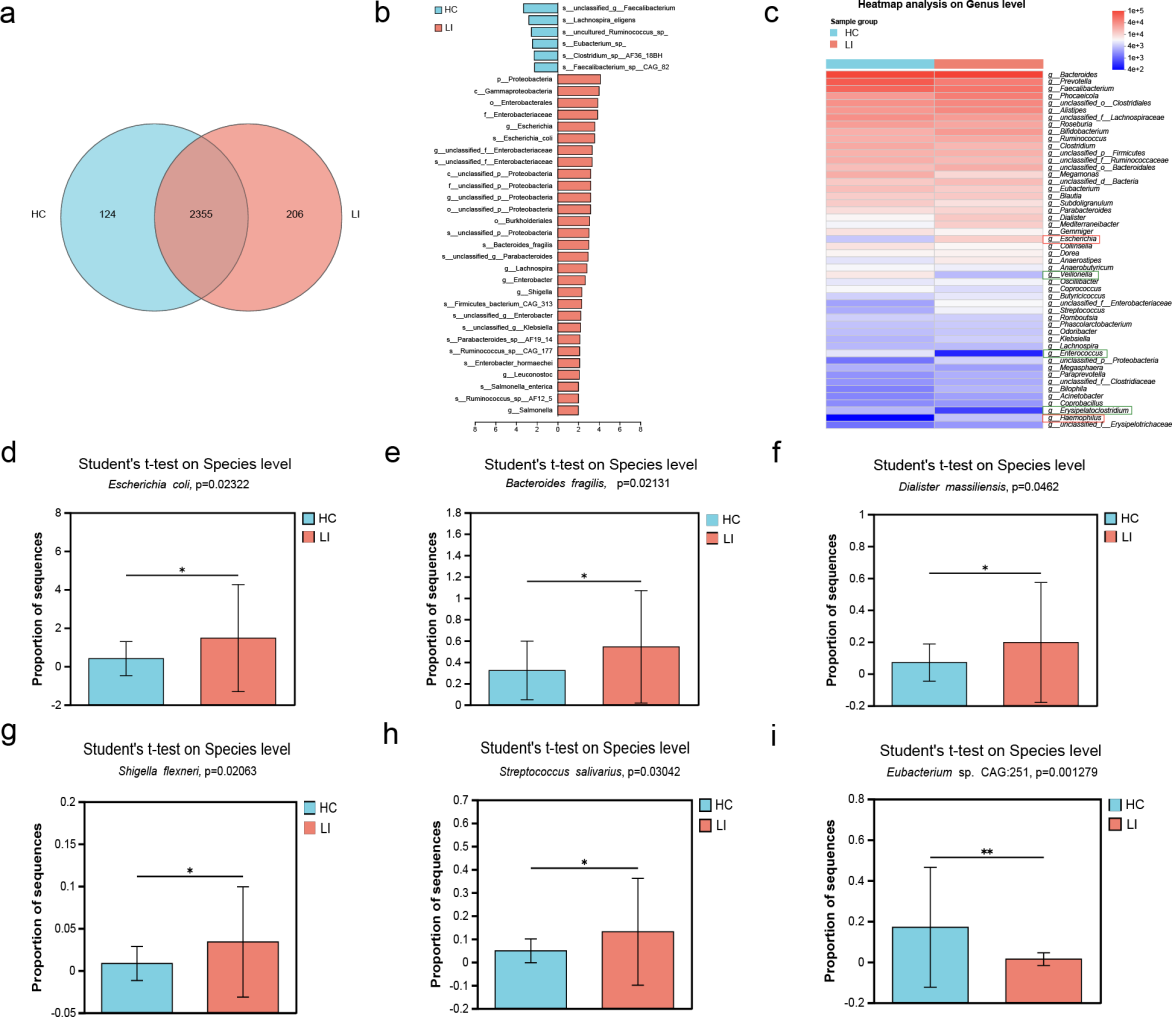
Differences in the gut microbial composition between Lis and HCs in the Chinese cohort. (a) Venn diagram analysis showing the total number of core species shared and unique. (b) LEfSe analysis reveals significant taxonomic differences between LI patients and HCs. (c) Heatmap based on the relative abundance of the genera between the two groups. Each row represents a species, and each column represents a sample/group. The blue-to-red gradient in the graph reflects the change in abundance from low to high. The closer to blue, the lower the abundance, and the closer to red, the greater the abundance. (**d–i**) Comparison of the relative abundances at the species level according to metagenomic sequencing between the two groups. The value of *P* indicates the parameter variance of all groups in the plot using the unpaired *t*-test.

**Fig 4:**
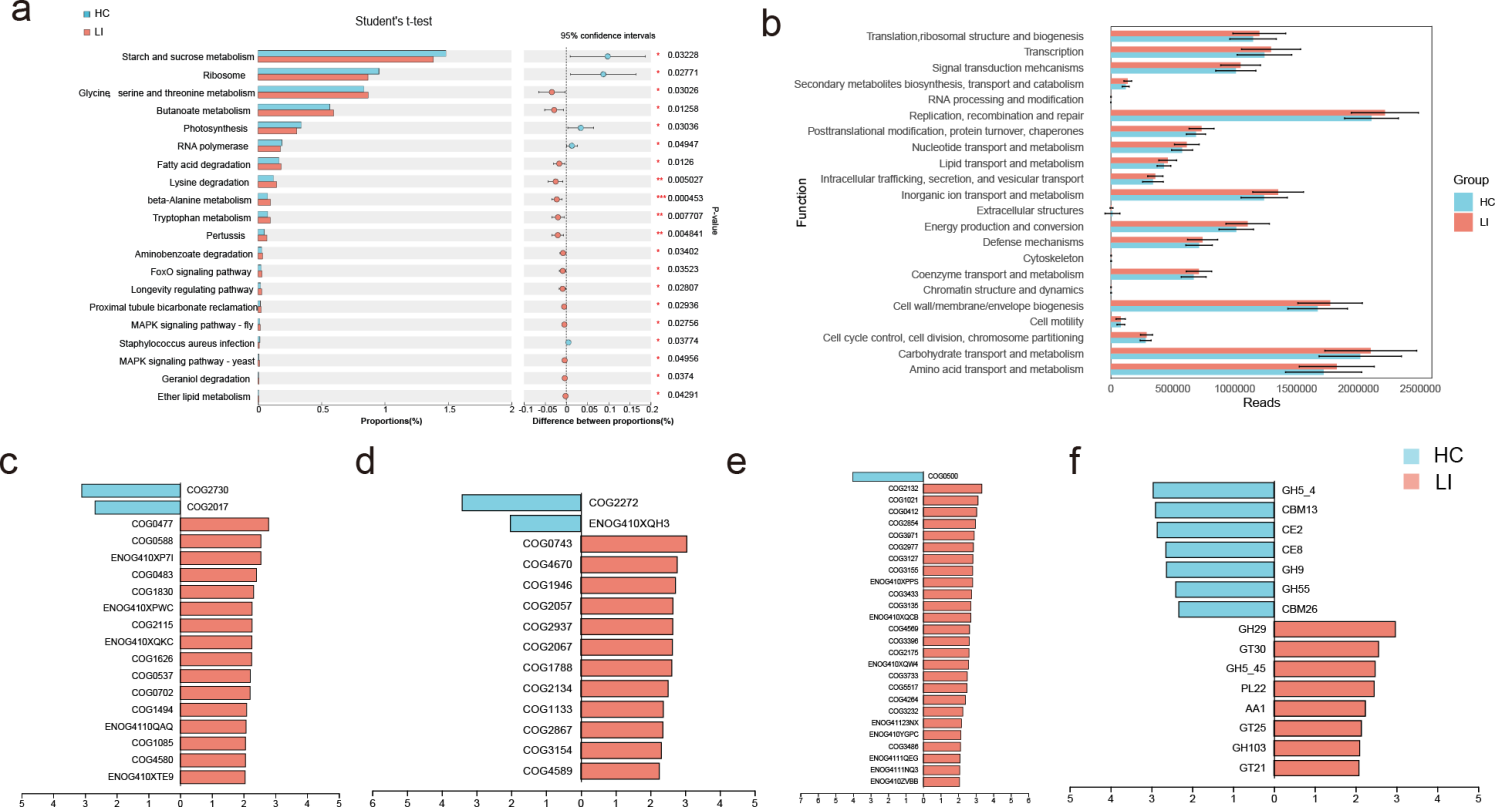
Comparison of microbial functions between the two groups. (a) Differences in KEGG pathways between the two groups. (b) The differential abundance of functional categories encoded by COG in the metagenome. Species associated with (c) carbohydrate transport and metabolism, (d) lipid transport and metabolism, (d) secondary metabolite biosynthesis, transport and catabolism, and genes encoding (**F**) carbohydrate-active enzymes (CAZy) based on LEfSe.

The Venn diagram (Fig. 3a) illustrates the shared and unique genera between the LI and HC groups. Among the two groups, 2,355 genera were shared, 124 genera belonged exclusively to the control group, and 206 genera were unique to the LI group. Furthermore, we observed substantial differences in the gut microbial profiles between the LI and HC groups at the phylum and species levels. According to the LEfSe analysis, at the phylum level, the relative abundance of Proteobacteria was enriched in the LI group, while the abundances of unclassified_g_Faecalibacterium and Lachnospira eligens were greater in the HC group (Fig. 3b; Table S3). Compositional analysis revealed significant alterations in microbial abundance at the genus and species levels between LI patients and HCs. At the genus level, LI patients showed a reduced abundance of *Veilonella*, *Enterococcus*, and *Erysipelatoclostridium*, while there was a greater abundance of *Escherichia* and *Haemophilus* in LI patients than in controls (Fig. 3c). At the species level, the abundances of *E. coli* (*P* = 0.023), *Bacteroides fragilis* (*P* = 0.021), *Dialister massiliensis* (*P* = 0.046), *Shigella flexneri* (*P* = 0.020) and *Streptococcus salivarius* (*P* = 0.030) decreased in HC patients (Fig. 3d through h). The only more abundant species in the HC group was *Eubacterium* sp. CAG:251 (*P* = 0.001) (Fig. 3i). Next, we investigated the roles of microorganisms and their harbored genes in the KEGG metabolic pathways essential for LI. We detected 15 upregulated and 5 downregulated pathways in the LI group compared with the control group; these pathways were primarily involved in amino acid metabolism, butanoate metabolism, and fatty acid degradation, among others (Fig. 4a). In addition, Fig. 4b shows the functional reads plotted by ImageGP (23), illustrating the differential abundance of functional categories encoded by COG in the metagenome. Interestingly, we identified several species whose functions included carbohydrate transport and metabolism (Fig. 4c); lipid transport and metabolism (Fig. 4d); and secondary metabolite biosynthesis and transport and catabolism (Fig. 4e) according to COG functional annotation; these species appeared to be highly abundant in the microbiome of the LI group. Furthermore, the total abundance of genes encoding CAZy, including GH29, GT30, GH5_45, PL22, AA1, GT25, GH103, and GT21, was significantly greater in the LI group than in the HC group (Fig. 4f).

**Fig 5:**
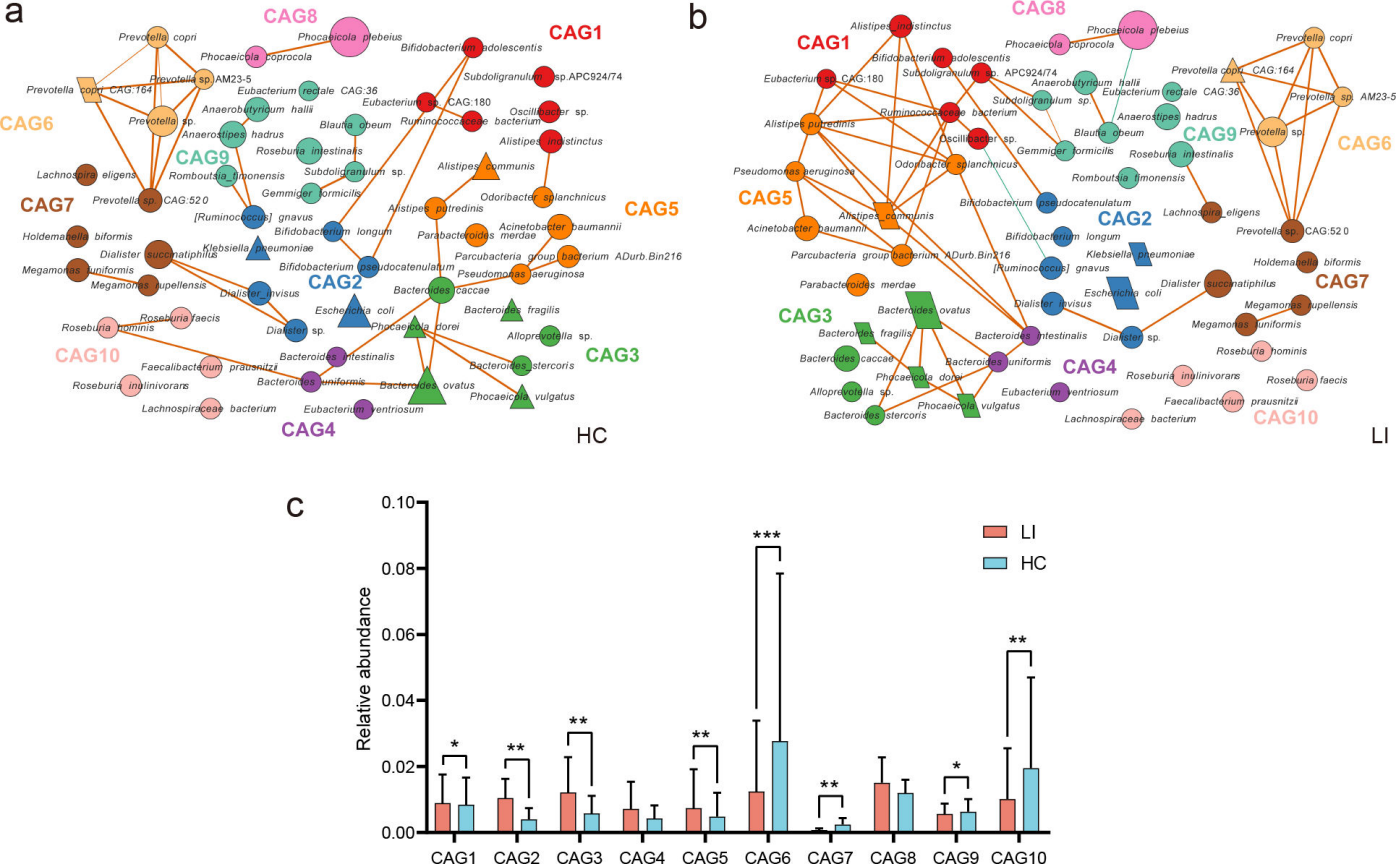
Species-level network diagram showing the enrichments in two groups based on significantly changed CAGs. Node size reflects the average abundance of each bacterium. Lines connecting nodes denote their correlations, with line thickness denoting correlation strength. Positive correlations are depicted in red, while negative correlations are shown in green. Only correlations with coefficients exceeding 0.4 are displayed. (a) The network of the HC group. (b) The network of the LI group. (c) The relative abundances of the 10 CAGs exhibiting significant differences between the two groups were determined using the Wilcoxon rank-sum test (*P < 0.05, **P < 0.01, ***P < 0.001).

In addition, we also investigated the formation of functional groups, also known as “guilds,” among bacteria in the gut ecosystem. These guilds interact with each other and have an impact on the health of the host (34). To compare the coabundance networks of microbiota between individuals with LI and HCs, we identified potential key species networks within the gut microbiota of LI patients. We constructed a coabundance network and clustered the 55 species into 10 CAGs. And then employed hierarchical clustering and WGCNA to identify potential guild structures within these CAGs (Fig. 5a and b). Each CAG consisted of 2–8 species. Notably, the expression of CAG6, CAG7, CAG9, and CAG10 was significantly lower in the LI patients than in the HCs, while the expression of CAG1, CAG2, CAG3, and CAG5 was significantly greater in the LI patients (Fig. 5c). Specifically, the bacteria belonging to CAG3, which are from the Bacteroidales order (such as *Alloprevotella* sp., *Bacteroides caccae*, *B. fragilis*, *Bacteroides ovatus*, *Bacteroides stercoris*, and *Phocaeicola dorei*), displayed a greater number of lines and larger nodes in the LI group than in the HC group. By contrast, CAG9 and CAG10, which demonstrated decreased relative abundances in the LI group, comprised 85% of bacteria from the Ruminococcaceae and Lachnospiraceae families, whose members have the potential to produce short-chain fatty acids (SCFAs) (Fig. 5b).

In addition, we constructed a coabundance network of genera and clustered the 54 genera into 10 CAGs within the identified LI patient group of AGP. Each CAG gene contained 2–10 genera. We observed a significant increase in the expression of six CAGs, namely, CAG1, CAG2, CAG3, CAG4, CAG6, and CAG7, in the LI group compared to the HC group. Similar to our case–control study, the relative abundance of CAG4, which is a genus of Bacteroides and Parabacteroides, increased in the LI group in the AGP ( Fig. S4).

### Associations between the gut microbiome and serum metabolites

To further investigate the association between the gut microbiome and serum metabolites and determine the potential contribution of these microbiome-associated metabolites in predicting LI, we conducted an integrated analysis of the microbiome and metabolome. We performed a comprehensive analysis of fecal and matched serum samples from a cohort of 28 LI patients and 28 HCs. The metagenomic profiles of the fecal samples were associated with the metabolome profiles of the serum samples.

**Fig 6:**
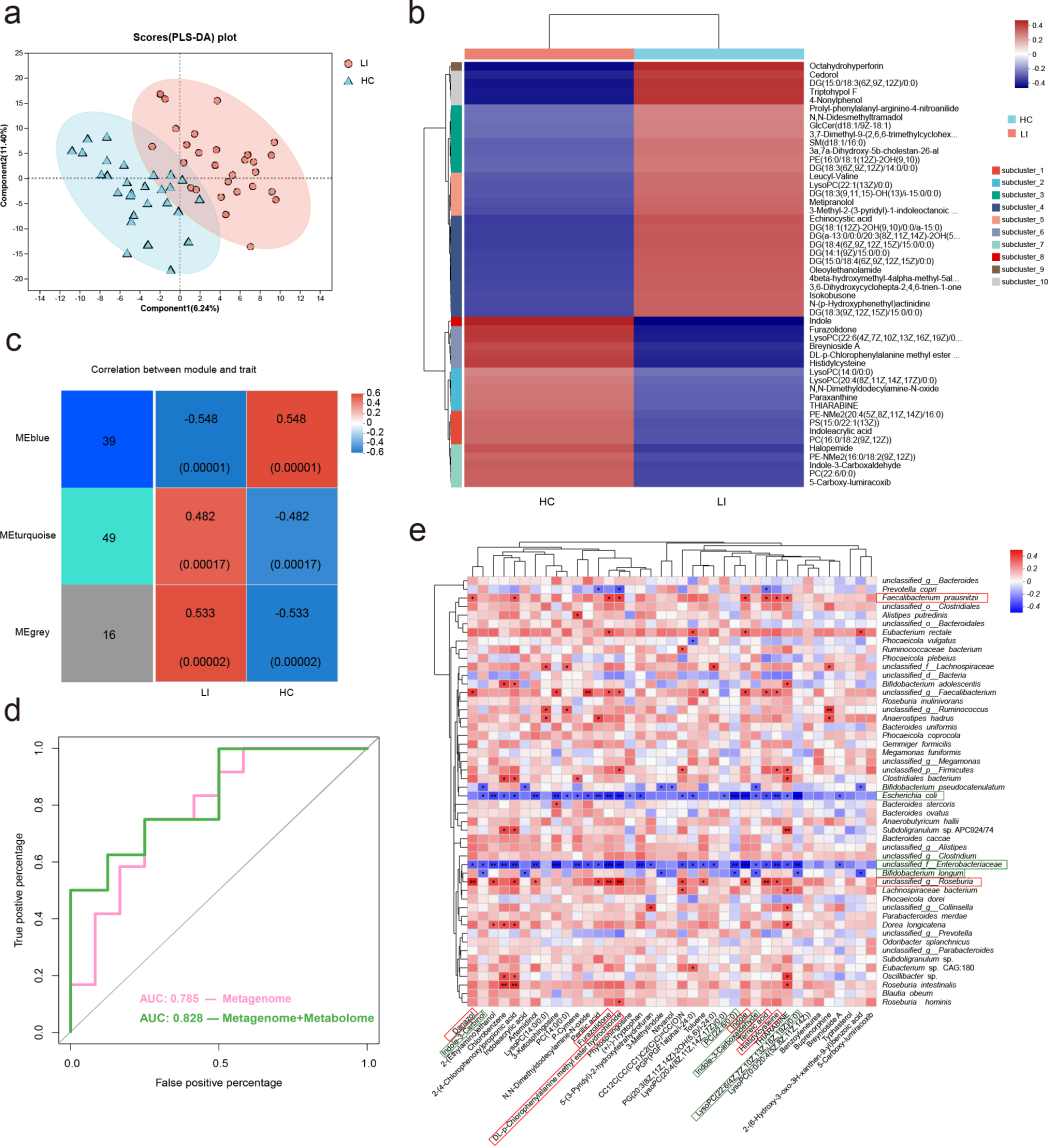
Associations of gut microbial species with circulating metabolites. (a) Supervised PLS-DA showing differences between LI patients and HCs. (b) Hierarchical cluster analysis of differentially abundant metabolites between HCs and LIs. (c) WGCNA coexpression network analysis. Spearman correlation analysis of the network modules with traits. After optimizing and merging the DEGs, the genes were divided into three modules: MEblue, MEturquoise, and MEgray. The color gradient indicates the direction, that is, positive (red) or negative (blue), and the strength of the correlation. (d) The AUC values of the serum metabolites combined with the microbiota (green) random forest model and the microbiota alone (pink) random forest model. (e) Heatmap of Spearman’s correlation between 39 discriminatory metabolites and the top 50 key bacterial species. The red squares indicate positive correlations, whereas the blue squares indicate negative correlations.

In the metabolomic analysis, we conducted nontargeted profiling of serum samples and identified 1,109 features based on retention time and mass/charge ratio. Among these, 104 features were detected in both positive and negative ionization modes (Table S4). Notably, the LI group exhibited pronounced metabolic alterations compared to the HCs. We employed a supervised partial least-squares discriminant analysis (PLS-DA) using two components (*R*^2^*X*_cum_ = 0.287, *R*^2^*Y*_cum_ = 0.99, *Q*_cum_^2^ = 0.594) to explore metabolic differences in serum metabolites. This analysis revealed some differences between the LI and HC groups (Fig. 6a). A total of 104 metabolites showed significant differences in abundance between the LI and HC groups, with 58 metabolites having higher concentrations and 47 metabolites having lower concentrations in the LI group (*P* < 0.05; variable importance in projection [VIP] value of 0.1 and fold change [FC] of 1) (Fig. 6b).

Furthermore, we performed a co-occurrence analysis of the serum metabolites using WGCNA. This analysis allowed us to group the 104 serum metabolites into three statistically significant modules (Fig. 6c). Among these three modules, module 1 displayed a positive correlation with LI subjects but a negative correlation with HCs. This suggests its potential significance as a key module associated with LI, supported by its highest correlation coefficient and the most significant *P* value among the three modules. This cluster comprised 39 metabolites, many of which were enriched in sphingolipid metabolism, tryptophan metabolism, and choline metabolism in cancer (Fig. S5).

In addition, we developed two random forest regression models using only microbial species and a combination of microbial species and serum metabolites. The models using microbial species with serum metabolites (AUC value of 0.828) outperformed those based on the gut microbiota alone (AUC value of 0.785) (Fig. 6d). The combined analysis of the top 35 bacterial species and 28 serum metabolites showed that a subset of seven bacterial species and nine serum metabolites (Table S5) had the highest discriminatory ability. We calculated Spearman’s correlation coefficients to assess the associations between the relative abundance of the 50 top species and the 39 key individual metabolomic features identified by WGCNA. We detected a positive correlation between the serum concentrations of probiotic species, such as *Faecalibacterium prausnitzii* and s_unclassified Roseburia, and higher concentrations of substances (e.g., danazol, furazolidone, DL-p-chlorophenylalanine methyl ester hydrochloride, indole, terreic acid, and histidylcysteine). Conversely, *E. coli*, s_unclassified Enterobacteriaceae, and *Bifidobacterium longum* exhibited inverse relationships with reduced levels of indole-3-carbinol, PC (22:6/0:0), indole-3-carboxaldehyde, and LysoPC (22:6(4Z,7Z,10Z,13Z,16Z,19Z)/0:0) (Fig. 6e).

### FMT animal study

**Fig 7:**
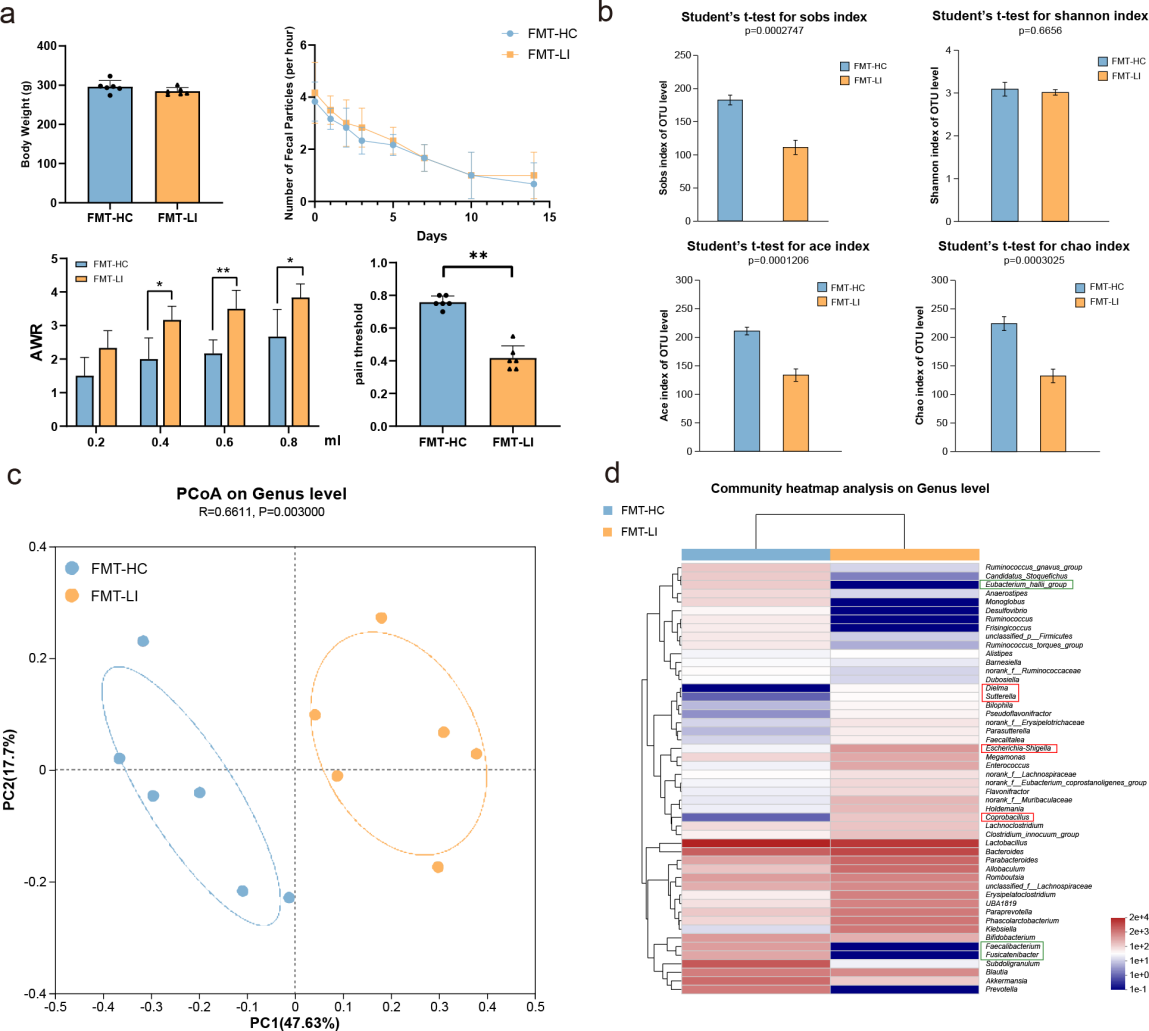
Overall structural variations in the bacterial diversity and microbial community composition of the gut microbiota between the FMT-HC and FMT-LI groups. (a) Differences in body weight, number of fecal particles, AWR, and pain threshold between the two groups. (b) The differences in α-diversity between the two groups were estimated by the Sobs, ACE, Shannon, and Chao1 indices using Student’s *t*-test. (c) Principal coordinate analysis based on β-diversity. The closer the distance between samples is, the more similar the species. (d) Heatmap based on the relative abundance of the genera between the two groups.

**Fig 8:**
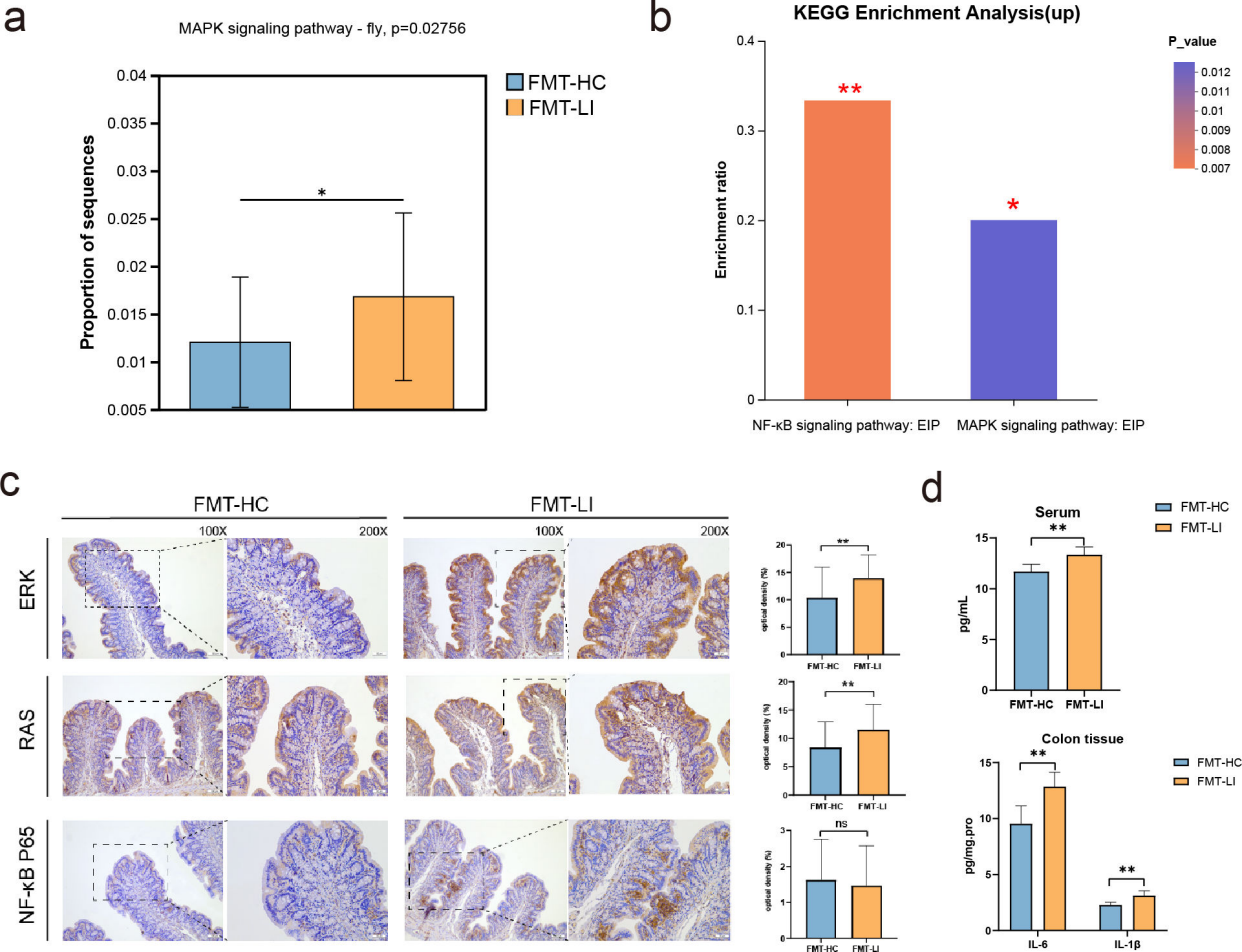
MAPK signaling is involved in LI pathophysiology. (a) MAPK signaling pathway enrichment based on gut metagenome and serum metabolomics using the KEGG database. (b) The NF-κB signaling pathway was enriched according to serum metabolomics. (c) Immunohistochemical results show that ERK, RAS, and NF-κB are important proteins involved in the MAPK pathway in colonic tissue between the two FMT groups. The right-side images are the high-magnification portions of the left-side images. Representative immunohistochemical and summary histograms showing ERK (upper right), RAS (middle right), and NF-κB (lower right) protein expression in the colon of the two groups. 100×, bar = 100 µm; 200×, bar = 50 µm. (d) Comparison of the concentrations of IL6 and IL1β in colonic tissue and of serum IL6 between the FMT-HC and FMT-LI groups (*P < 0.05, **P < 0.01).

To assess the causal relationship between the gut microbiota and susceptibility to LI, a study in which gut bacteria from both LI patients and healthy individuals were transplanted into antibiotic-treated rats was conducted. In the absence of an external stimulus, rats receiving gut microbiota from LI patients (referred to as the FMT-LI group) exhibited greater levels of AWR and a lower pain threshold than those in the control group (referred to as the FMT-HC group). However, there was no significant change observed in terms of body weight or the number of fecal pellets (Fig. 7a). In addition, 16S rRNA sequencing analysis of gut microbial diversity, including the Sobs, Shannon, ACE and Chao1 indices, revealed a notable difference between the two groups (Fig. 7b). Furthermore, beta diversity analysis using the Bray‒Curtis algorithm revealed significant differences in the microbiota composition at the genus level between the two groups (Fig. 7c). The abundances of the microbial genera *Faecalibacterium*, *Fusicatenibacter*, and *Eubacterium hallii* were significantly decreased, while others, such as *Escherichia, Shigella*, *Dielma*, *Sutterella*, and *Coprobacillus*, were more abundant in the FMT-LI group than in the FMT-HC group, similar to the findings from the LI metagenome data (Fig. 7d). KEGG pathway analysis revealed that the MAPK signaling pathway was enriched in the gut microbiota and serum metabolites associated with LI (Fig. 8a). Previous research has shown that the NF-κB signaling pathway, a classic inflammatory signaling pathway, interacts with the MAPK signaling pathway (35–37). In addition, the NF-κB signaling pathway was significantly enriched in the LI-related serum metabolites (Fig. 8b). Therefore, we investigated the expression of NF-κB, ERK, and RAS in the colonic tissue of both the FMT-LI and FMT-HC groups using immunohistochemistry. The results showed that the expression of ERK and RAS in the FMT-LI group was significantly greater than that in the FMT-HC group, but there were no significant differences in NF-κB expression between the two groups (Fig. 8c). Moreover, colonic tissue exhibited elevated concentrations of the proinflammatory cytokines IL-6 and IL-1β, and the serum levels of IL-6 were significantly greater in the FMT-LI group than in the control group (Fig. 8d). Thus, these data suggest that the gut microbiota associated with LI is involved in inflammation pathways.

## DISCUSSION

Lactose intolerance is widely recognized as resulting from decreased production of lactase (encoded by a variant of the lactase LCT gene), which typically occurs after weaning during childhood (1). Although LI is prevalent in China due to a high level of LNP (6), it is important to note that many people have not been identified as having LI. This suggests that lactase deficiency may not be the only cause of LI. Our study demonstrated that dysbiosis of the gut microbiota and associated changes in host metabolism may contribute to the pathophysiology of LI. First, we conducted a paired-sample study using data sets from the AGP to assess differences in gut microbial composition between individuals with LI and those without LI. Next, we employed metagenomic sequencing and serum metabolome analysis to investigate the characteristics of the gut microbiota and the links between the gut microbiota and serum metabolome composition in LI patients and HCs from China. To the best of our knowledge, this bioinformatics analysis is the first to utilize a publicly available database and examine metagenomic and metabolomic signatures by recruiting patients with lactose intolerance. Finally, our FMT animal study further confirmed changes in the gut microbiota, visceral hypersensitivity, and inflammatory responses in the LI groups.

Here, we observed a significant reduction in fecal microbiota α-diversity (ASV richness, Chao1 index, and ACE index) in the LI group compared to the non-LI group in the AGP study. However, in our case‒control study, there was no difference in the α- diversity of the gut bacterial communities between the LI group and HCs. Similarly, the results of 16S rRNA sequencing showed that LEfSe analysis revealed 23 taxa that differed between LIs and non-LIs, and the metagenome results showed that 29 taxa differed between LIs and HCs. The gut microbiota of the LI group in our case‒control study was characterized by high levels of Proteobacteria, while SR1 was significantly more abundant in LI patients with AGP. Both of these phyla are associated with harmful bacteria and inflammatory responses. Previous studies have provided evidence that specific gut symptoms experienced by LI patients might be the result of an abundance of Bifidobacterium in the gut (38). Although we did not find increased Bifidobacterium abundance in the LI group in either of the two cohorts, the abundances of *Parabacteroides* and *B. fragilis*, which belong to the Bacteroidales order, were more abundant in the LI group than in the HC group. Interestingly, we found higher levels of Dialister and lower levels of Eubacterium in the LI group than in the HC group, both in the Western cohort and the Chinese cohort. In addition, the species that were most enriched in LI patients in the Chinese cohort included *B. fragilis* and *E. coli*, most of which are pathogenic bacteria (39). Functionally, KEGG pathway analysis based on metagenomics revealed that gut dysbiosis in the LI was closely linked to a decrease in starch and sucrose metabolism and an increase in glycine, serine, butanoate metabolism, and fatty acid degradation. Similarly, our PICRUSt2 analyses of the AGP showed that the pathways differentially expressed between the two groups were mainly involved in the increase in homolactic fermentation, the pentose phosphate pathway, and glycolysis, which are also related to carbonate metabolism.

Ecologically, gut bacteria function as functional groups and coexist in the human gut microbiota, and interactions among them could be linked to health and disease (40). Therefore, in our study, we created coabundance networks based on correlations to study the interactions between microbes, facilitating the identification of functionally important members of the intestinal microbiota. Here, we found a significant difference in the abundance of CAG between the two groups. This CAG exhibited enrichment of Bacteroides, which demonstrated increased strength and interaction within the LI group. Bacteroides is considered an important primary degrader of complex nondigestible carbohydrates, which generate oligosaccharides, which, in turn, can be fermented by secondary degraders (41). Many studies have demonstrated that the genus Bacteroides can metabolize complex polysaccharides, producing extracellular oligosaccharides. These oligosaccharides may be shared with other microbial members in the gut, including certain species of Bifidobacterium, which can metabolize a variety of oligomeric and simple carbohydrates (42).

In a previous study (43), it was observed that the intolerant group had significantly greater production rates of D- and L-lactate, acetate, propionate, and butyrate than did the tolerant group following lactose fermentation *in vitro*. Our results may clarify this phenomenon due to the greater interaction of Bacteroides in the LI group. However, unlike our study, in our previous study, the fecal bacterial composition did not differ between the two groups. We believe that this discrepancy may be due to differences in sample size and the methodology used for detecting the gut microbiota between the two studies. Another study also demonstrated clear differences in fermentation patterns between nonlactose digesters and lactose malabsorbers with intolerance (44).

Our metagenome results suggest that the differential composition of the gut microbiota is responsible for these distinct fermentation abilities, particularly the increased presence of CAG6, which indicates a greater ability to ferment carbohydrates.

Metabolomic analyses of blood samples revealed a distinctive distribution of several metabolites in individuals with LI. In this regard, we found that the levels of 39 serum metabolites, which are considered the key metabolites according to WGCNA, decreased. The identification of an LI biomarker involved a composite of nine serum metabolites and seven specific bacterial species, which significantly enhanced the discriminative capability of the LI (AUC = 0.828). Furthermore, the abundance of *E. coli*, a highly discriminatory species predicted by random forest analysis, was negatively correlated with the serum levels of indole and tryptophan. Previous studies (45) have demonstrated the role of the gut microbiota, including *E. coli*, *Proteus vulgaris*, *Paracolobactrum coliforme*, *Achromobacter liquefaciens*, and *Bacteroides* spp., in the transformation of tryptophan into indole and its derivatives. In our study, KEGG pathway enrichment analysis of the metagenome revealed greater tryptophan metabolism in the LI group than in the HC group, which corresponded to an increased abundance of *E. coli*. However, the serum levels of tryptophan and indole significantly decreased in the LI group. Similarly, bacterially derived tryptophan metabolites such as indoles and indole propionic acid (IPA) were also found to be lower in patients with IBD and type 2 diabetes than in HCs (46, 47). Tryptophan metabolites not only support the differentiation and function of anti-inflammatory macrophages, Treg cells, CD4 + CD8αα immune regulatory cells, and IL-10 and/or IL-35 B regulatory cells but also foster the development of IL-22-producing ILC3s. These metabolites are crucial for maintaining gut and systemic homeostasis (48). PGF1alpha, a stable metabolite of prostaglandin I2 (PGI2), plays a crucial role in an integrated network with various pharmacological effects, including vasodilation, inhibition of smooth muscle cell proliferation, and platelet aggregation (49). Our study revealed that PGF1alpha is downregulated and negatively correlated with the abundance of *E. coli* in the LI. Endogenous PGI2 has inhibitory effects on immune responses against pathogens and allergens (50). Furthermore, a decrease in the abundance of *Eubacterium rectale* was observed in both the Chinese and American LI groups. This decrease showed a positive correlation with PGF1alpha and an inverse correlation with indole, indicating a potential therapeutic role for this bacterium in the treatment of LI.

In the FMT animal experiment, we observed higher AWR scores and a lower pain threshold in the FMT-LI group, indicating visceral hypersensitivity in the FMT-LI group, consistent with previous studies (51). Furthermore, we found significant differences in the α- and β-diversity between the FMT-LI and FMT-HC groups. The abundance of harmful bacteria, such as Escherichia and Shigella, increased significantly, while the abundance of beneficial bacteria, such as butyrate producers such as *F. prausnitzii*, decreased significantly. These findings align with the results observed in LI patients. Based on the enrichment analysis of the serum metabolome and microbiota metagenomic data in the LI group, the MAPK signaling pathway was identified as being commonly enriched. To verify the role of the MAPK pathway, we focused on RAS, ERK, and NF-κB, which are key molecules in the MAPK signaling pathway, using the FMT animal model. Our results showed higher expression levels of ERK, RAS, and proinflammatory cytokines in the FMT-LI group than in the control group, indicating an increase in the inflammatory response. The MAPK pathway is involved in various biological functions, such as proliferation, gene expression, differentiation, mitosis, cell survival, apoptosis, and cellular metabolism in cancer cells (52). Although there are conflicting results regarding the association between LI and common cancer types, such as colorectal, ovarian, and prostate cancers (53–56), some studies support a correlation between lactose consumption or LI and cancers (57). This may explain the enrichment of the MAPK signaling pathway observed in the LI group.

The present study has several limitations. First, in the analysis using the AGP data set, it remains unclear whether lactase phenotypes (lactase persistence and lactase nonpersistence) are associated with LI or non-LI individuals. In addition, we did not investigate the potential impact of diet, particularly milk consumption, on the gut microbiota in the Chinese cohort. Future studies should include larger cohorts with lactose intolerance to address these gaps.

In this study, we identified significant changes in the gut microbiome that were accompanied by alterations in specific metabolites, such as those of *Escherichia* sp. CR1 and *Enterobacter* sp. FY.07, indole, PGF1alpha, and the MAPK signaling pathway, were found to be involved in the LI group. Our multiomics study and animal experiments have provided valuable insights into the dysbiosis of microbial function and metabolism in the LI, providing crucial information for the development of preventive and therapeutic strategies.

## Data Availability

The data sets generated and/or analyzed during the current study are available in the NCBI repository, PRJNA1082417 and PRJNA1125135.

## SUPPLEMENTAL MATERIAL

Fig. S1 msystems.00839-24-s0001.tif Demographic profile comparison between 562 patients self-reporting LIs and an equal number of 562 self-reporting non-LIs.

Fig. S2 msystems.00839-24-s0002.tif A prospective machine learning model for identifying LIs.

Fig. S3 msystems.00839-24-s0003.tif Variations in gut microbiota diversity between the two cohorts.

Fig. S4 msystems.00839-24-s0004.tif Genus-level coabundance network diagram in the AGP showing the enrichments in two groups based on significantly differential CAGs.

Fig. S5 msystems.00839-24-s0005.tif The 39 metabolites within the MEblue module were enriched in pathways related to sphingolipid metabolism, tryptophan metabolism, and choline metabolism in cancer.

Legends msystems.00839-24-s0006.docx Supplemental figure and table legends.

Supplemental Tables msystems.00839-24-s0007.xlsx Tables S1 to S5.

ASM does not own the copyrights to Supplemental Material that may be linked to, or accessed through, an article. The authors have granted ASM a non-exclusive, world-wide license to publish the Supplemental Material files. Please contact the corresponding author directly for reuse.
