# Supplementary figures and images for: Gut microbiome and serum metabolome alterations associated with lactose intolerance (LI): a case‒control study and paired-sample study based on the American Gut Project (AGP)
Source: mSystems. 2024 Sep 25;9(10):e00839-24. doi: 10.1128/msystems.00839-24 (PMC11494873; doi:10.1128/msystems.00839-24)

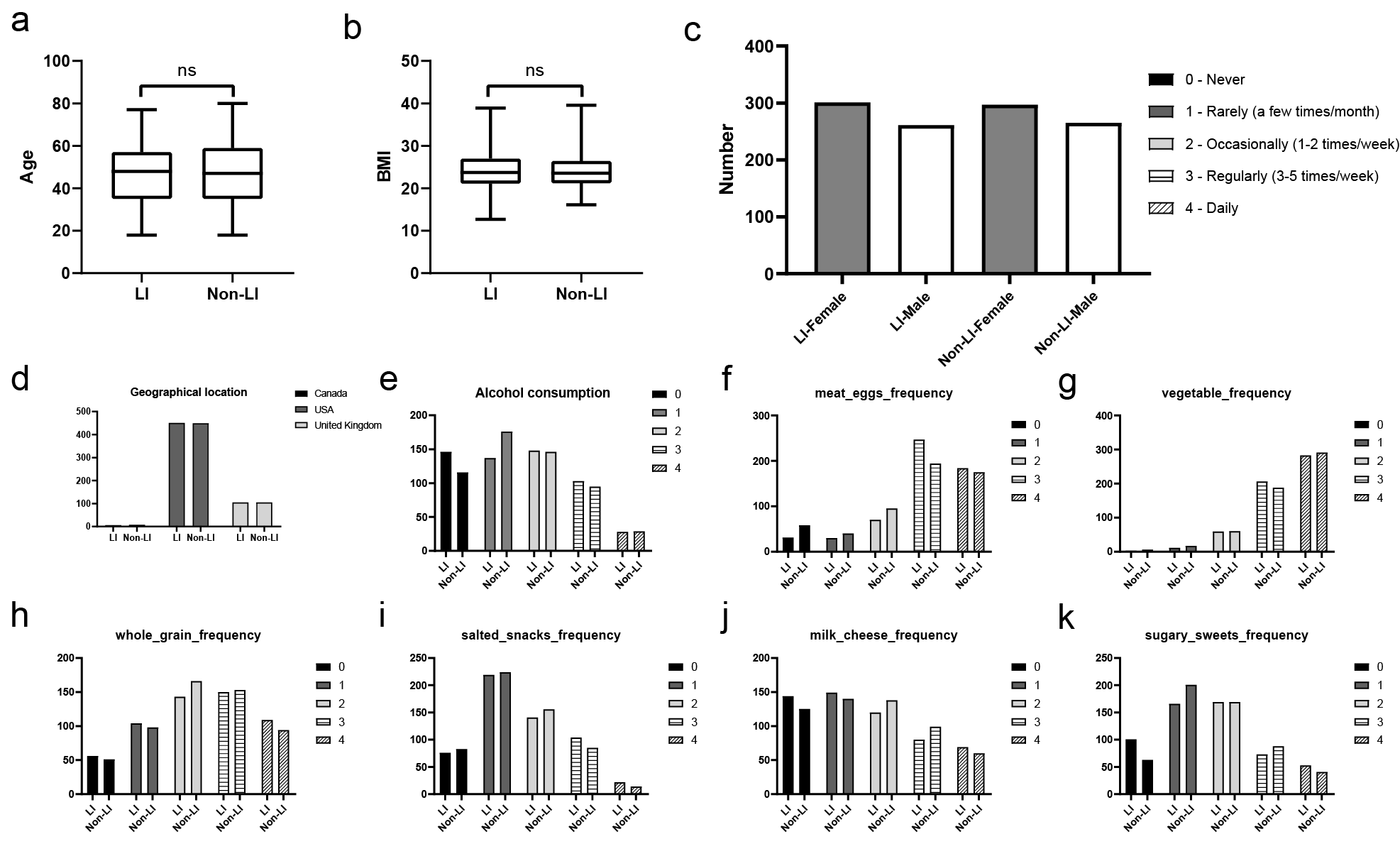

Supplement: Fig. S1 — Demographic profile comparison between 562 patients self-reporting LIs and an equal number of 562 self-reporting non-LIs. [file msystems.00839-24-s0001.tif]

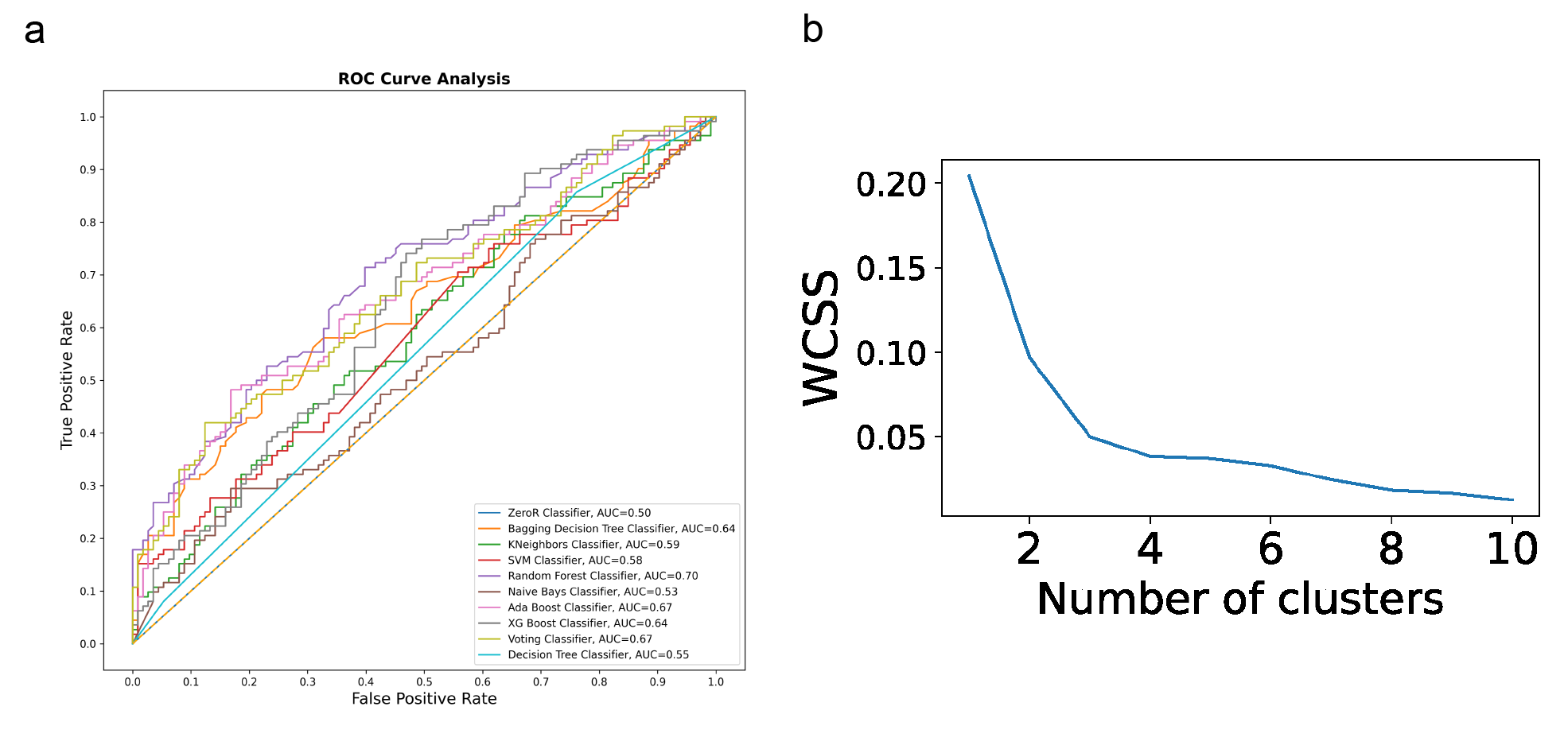

Supplement: Fig. S2 — A prospective machine learning model for identifying LIs. [file msystems.00839-24-s0002.tif]

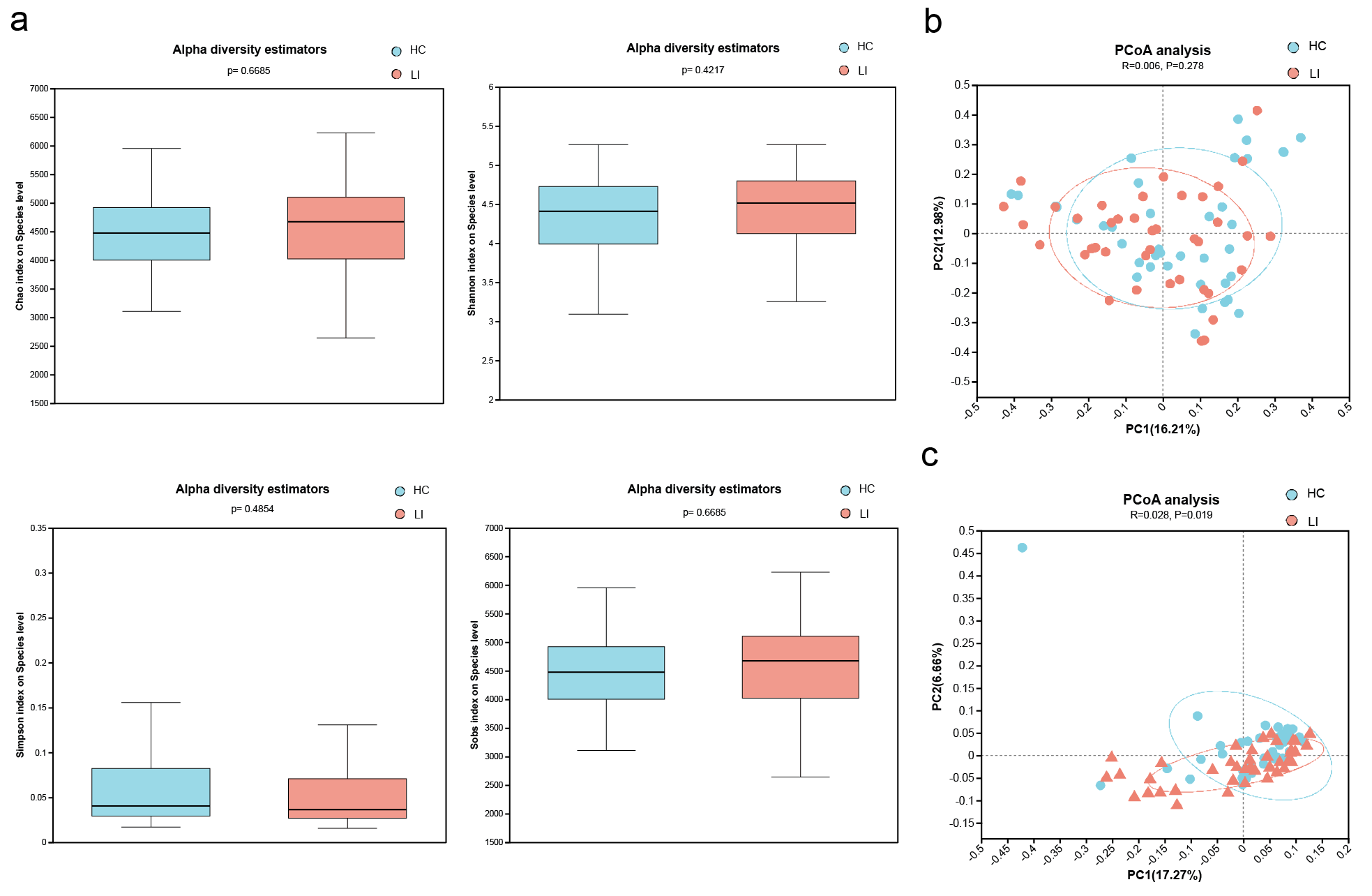

Supplement: Fig. S3 — Variations in gut microbiota diversity between the two cohorts. [file msystems.00839-24-s0003.tif]

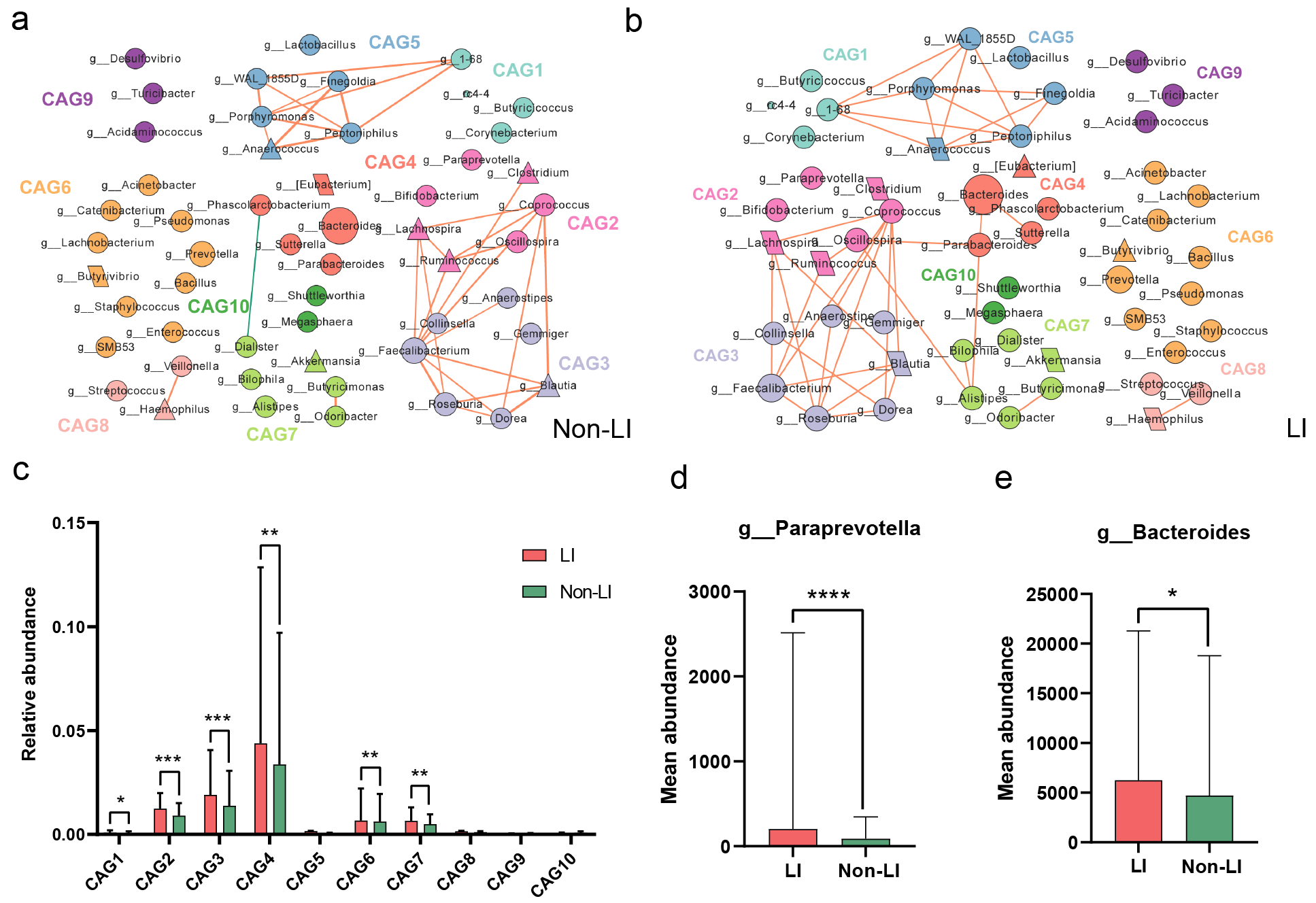

Supplement: Fig. S4 — Genus-level coabundance network diagram in the AGP showing the enrichments in two groups based on significantly differential CAGs. [file msystems.00839-24-s0004.tif]

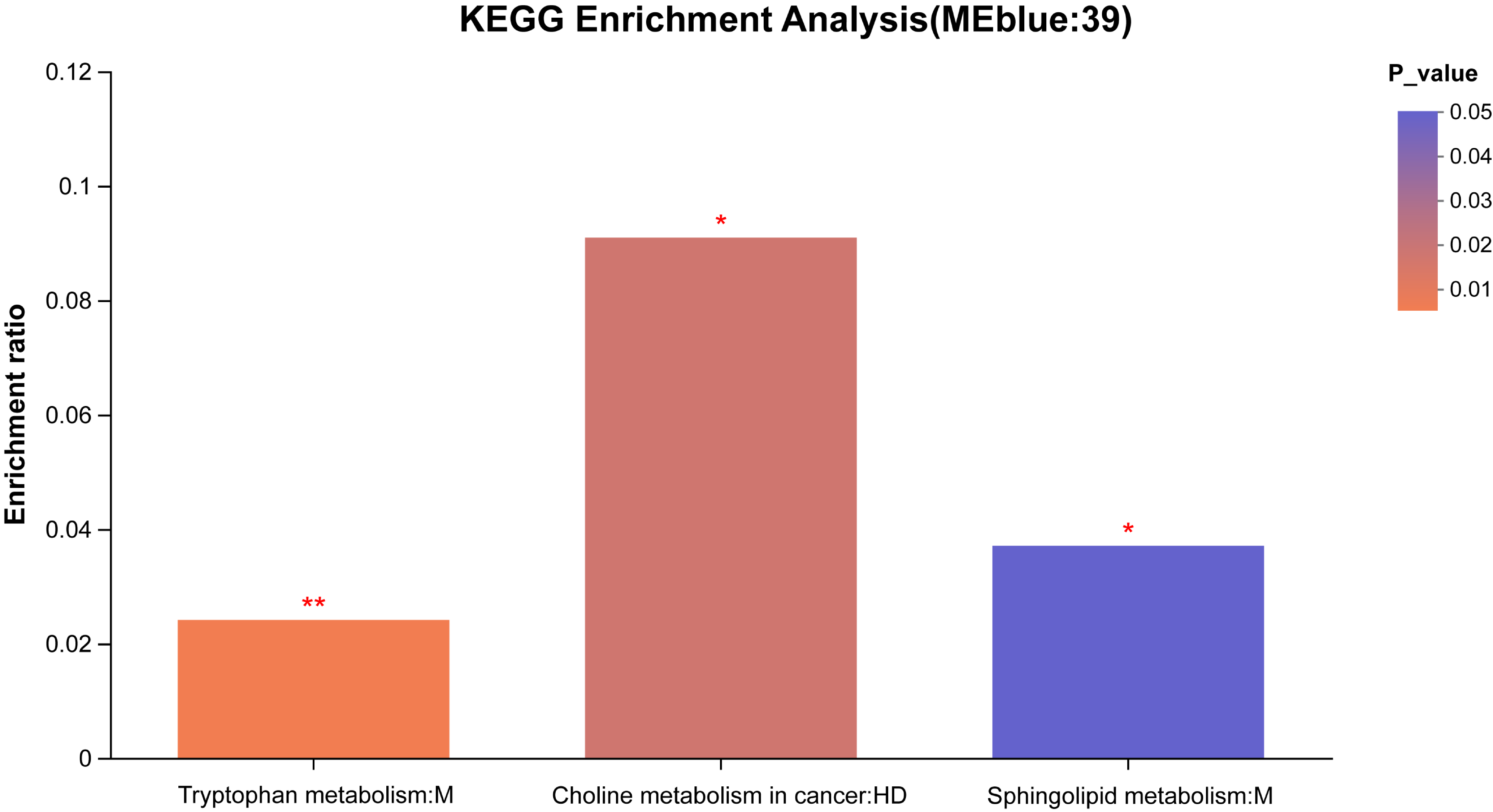

Supplement: Fig. S5 — The 39 metabolites within the MEblue module were enriched in pathways related to sphingolipid metabolism, tryptophan metabolism, and choline metabolism in cancer. [file msystems.00839-24-s0005.tif]
