# Supplementary material for: Gut microbiome and serum metabolome alterations associated with lactose intolerance (LI): a case‒control study and paired-sample study based on the American Gut Project (AGP)
Source: mSystems. 2024 Sep 25;9(10):e00839-24. doi: 10.1128/msystems.00839-24 (PMC11494873; doi:10.1128/msystems.00839-24)
Supplement: Legends — Supplemental figure and table legends. [file msystems.00839-24-s0006.docx]

**Supplementary Figure legends**

Figure S1 Demographic profile comparison between 562 patients self-reporting LIs and an equal number of 562 self-reporting non-LIs. No disparities were observed in (a) age, (b) BMI, (c) sex between LIs and their matched controls, (d) geographical location, (e) alcohol consumption, (f) meat/egg frequency, (g) vegetable frequency, (h) whole grain frequency, (i) salted snack frequency, or (k) sugary sweet consumption frequency.

Figure S2 A prospective machine learning model for identifying LIs. (a) The performance of ten machine learning models assessed by their AUC values. (b) The optimal number of clusters identified by the elbow method.

Figure S3 Variations in gut microbiota diversity between the two cohorts. (a) The alpha diversity (Chao, Shannon, Simpson, and Sobs indices) of the gut microbiota in patients with LI and HCs. (b) PCoA based on Bray‒Curtis distances at the taxonomic level of the NR. (c) PCoA conducted utilizing the binary Jaccard algorithm on COG functional genes.

Figure S4 Genus-level coabundance network diagram in the AGP showing the enrichments in two groups based on significantly differential CAGs. Node size reflects the average abundance of each bacterium. Lines connecting nodes denote their correlations, with line thickness denoting correlation strength. Positive correlations are depicted in red, while negative correlations are shown in green. Only correlations with coefficients exceeding 0.4 are displayed. (a) The network of the HC group. (b) The network of the LI group. (c) The relative abundances of the 10 CAGs exhibiting significant differences between the two groups were determined using the Wilcoxon rank-sum test. (*P < 0.05, **P < 0.01, ***P < 0.001). The LI group in the AGP exhibited an elevated interaction between (d) g_Bacteroides and (e) g_Parabacteroides.

Figure S5 The 39 metabolites within the MEblue module were enriched in pathways related to sphingolipid metabolism, tryptophan metabolism, and choline metabolism in cancer.

Table S1 Comprehensive list of the host variables of the AGP cohort.

Table S2 The detailed characteristics of 41 patients with LI and 41 HCs.

Table S3 Bacterial distinctions at the species level between individuals with LI and HCs as determined by LEfSe analysis. (LDA>2, P<0.05)

Table S4 The abundance of 104 metabolites exhibiting significant differences between the LI group and the HC group.

Table S5 Potential biomarkers of microbial and metabolic markers for detecting patients with LI.
